# Chromosome doubling increases *PECTIN METHYLESTERASE 2* expression, biomass, and osmotic stress tolerance in kiwifruit

**DOI:** 10.1093/plphys/kiae475

**Published:** 2024-09-09

**Authors:** Yanyan Zhu, Xinlei Wang, Yan He, Yajing Liu, Runze Wang, Yongsheng Liu, Songhu Wang

**Affiliations:** Anhui Province Key Laboratory of Horticultural Crop Quality Biology, School of Horticulture, Anhui Agricultural University, Hefei, 230036, China; Anhui Province Key Laboratory of Horticultural Crop Quality Biology, School of Horticulture, Anhui Agricultural University, Hefei, 230036, China; Anhui Province Key Laboratory of Horticultural Crop Quality Biology, School of Horticulture, Anhui Agricultural University, Hefei, 230036, China; Anhui Province Key Laboratory of Horticultural Crop Quality Biology, School of Horticulture, Anhui Agricultural University, Hefei, 230036, China; Anhui Province Key Laboratory of Horticultural Crop Quality Biology, School of Horticulture, Anhui Agricultural University, Hefei, 230036, China; Anhui Province Key Laboratory of Horticultural Crop Quality Biology, School of Horticulture, Anhui Agricultural University, Hefei, 230036, China; Ministry of Education Key Laboratory for Bio-resource and Eco-environment, College of Life Science, State Key Laboratory of Hydraulics and Mountain River Engineering, Sichuan University, Chengdu, 610064, China; Anhui Province Key Laboratory of Horticultural Crop Quality Biology, School of Horticulture, Anhui Agricultural University, Hefei, 230036, China

## Abstract

Chromosome doubling-induced polyploidization is a popular tool for crop breeding. Polyploidy crops commonly have multiple advantages, including increased biomass and stress tolerance. However, little is known about the genes responsible for these advantages. We found kiwifruit (*Actinidia chinensis* cv. Hongyang) *PECTIN METHYLESTERASE 2* (*AcPME2*) is substantially upregulated in artificially created tetraploid plants that show increased biomass and enhanced tolerance to osmotic stress. Overexpression (OE) of *AcPME2* led to increased biomass and enhanced stress tolerance in Arabidopsis (*Arabidopsis thaliana*), tomato (*Solanum lycopersicum*), and kiwifruit. Upon short-term osmotic stress treatment, AcPME2-OE plants showed higher levels of demethylesterified pectins and more Ca^2+^ accumulation in the cell wall than Col-0 plants, which led to increased cell wall stiffness. The stress-induced plasmolysis assays indicated that AcPME2 dynamically mediated the cell wall stiffness in response to osmotic stress, which is dependent on Ca^2+^ accumulation. Transcriptomic analysis discovered that dozens of stress-responsive genes were significantly upregulated in the AcPME2-OE plants under osmotic stress. Besides, AcPME2-mediated cell wall reinforcement prevented cell wall collapse and deformation under osmotic stress. Our results revealed a single gene contributes to two advantages of polyploidization (increased biomass and osmotic stress tolerance) and that AcPME2 dynamically regulates cell wall stiffness in response to osmotic stress.

## Teaser

Accumulation of a pectin methylesterase that dynamically regulates cell wall stiffness increases biomass and osmotic stress tolerance in Arabidopsis, tomato, and kiwifruit.

## Introduction

Kiwifruit (*Actinidia chinensis*) is a woody perennial with fruits consumed worldwide. Kiwifruit provides multiple compounds including high contents of vitamin C, amino acid, minerals, and other metabolites beneficial to human health. The hybridization breeding of kiwifruit is time-consuming and difficult, due to its long juvenility and cross cycles (Varkonyi-Gasic et al. 2019). The polyploidy breeding has been successfully applied in kiwifruit. Colchicine-induced chromosome doubling in kiwifruit could increase cell size, plant biomass, and fruit size (Wu et al. 2011, 2012; Li et al. 2019; Zhu et al. 2021).

Whole genome duplication, which is achieved by spontaneous or artificial chromosome doubling, leads to polyploidization and plays an important role in plant evolution (Parisod et al. 2010; Bomblies and Madlung 2014; Chalhoub et al. 2014) and crop breeding (Renny-Byfield and Wendel 2014; Sattler et al. 2016; Touchell et al. 2020). As compared with diploid plants, polyploid plants commonly display multiple advantages including the increased biomass yield (Cai et al. 2007; Renny-Byfield and Wendel 2014; Vunsh et al. 2015; Corneillie et al. 2019; Zhu et al. 2021), the improved quality traits (Chen et al. 2018; Manzoor et al. 2019; Zhao et al. 2022), and the enhanced stress tolerance (Saleh et al. 2008; Oustric et al. 2017; Rao et al. 2020; Fatiukha et al. 2021; Wang et al. 2021; Alvarez-Morezuelas et al. 2022). Although several reports provided some clues for understanding a single advantage (Meng et al. 2016; Rao et al. 2020; Wang et al. 2021), little is known about the gene that simultaneously contributes to two advantages, such as both increased biomass yield and enhanced stress tolerance.

Plant cell wall plays a crucial role in cell division and expansion, plant growth, development, and interaction with the environment (Carpita and Gibeaut 1993). Pectin is one of major polysaccharides accumulated in the primary cell wall (Varner and Lin 1989) and can be modified by pectin methylesterases (PMEs), which cause the demethylesterification of homogalacturonans (Micheli 2001). When acting in a random way, PME-mediated demethylesterification contributes to cell wall loosening and cell extension by making homogalacturonans (HGs) available for HG-modifying enzymes, such as pectate lyases (Colin et al. 2023), or providing the platforms that anchor peroxidases to remodel plant cell wall (Moustacas et al. 1991; Francoz et al. 2019). Defects of *PME* genes can lead to a significant decrease in cell size of embryo (Levesque-Tremblay et al. 2015), in seed germination (Scheler et al. 2015), in root elongation (Wen et al. 1999), in pollen tube growth (Jiang et al. 2005), and in plant biomass (Stefanowicz et al. 2021). *PMEs* are also required for fruit softening (Wakabayashi et al. 2003; Wen et al. 2020; Xue et al. 2020). On the other hand, when acting in a linear way, PME-mediated demethylesterification contributes cell wall stiffening by producing continuous blocks of free carboxyl groups that could interact with calcium (Ca^2+^) to form a pectate gel (Micheli 2001; Willats et al. 2001). For instance, *PME35*-mediated demethylesterification provides mechanical support to the Arabidopsis (*Arabidopsis thaliana*) stem (Hongo et al. 2012). Some studies indicated that the action pattern of PME is dependent on the initial degree of pectin methylesterification and pH (Catoire et al. 1998; Denes et al. 2000; Micheli 2001). In addition, PMEs mediate plant tolerance to heat stress (Wu et al. 2010; Huang et al. 2017) and high salinity (Yan et al. 2018; Gigli-Bisceglia et al. 2022).

Our previous study indicated that several *PME* genes were significantly upregulated in the colchicine-induced tetraploid *Actinidia chinensis* var. “Hongyang” (Zhu et al. 2021). It remains to be determined whether the upregulation of *PMEs* contribute to the increased cell size and biomass of tetraploid kiwifruit. In this study, we revealed that overexpression of *AcPME2* led to substantially increased biomass and osmotic stress tolerance in Arabidopsis, tomato (*Solanum lycopersicum*), and kiwifruit. AcPME2 dynamically mediated cell wall stiffness in response to osmotic stress.

## Results

### Chromosome doubling increased the biomass and tolerance to osmotic stress in kiwifruit

**Figure 1.**
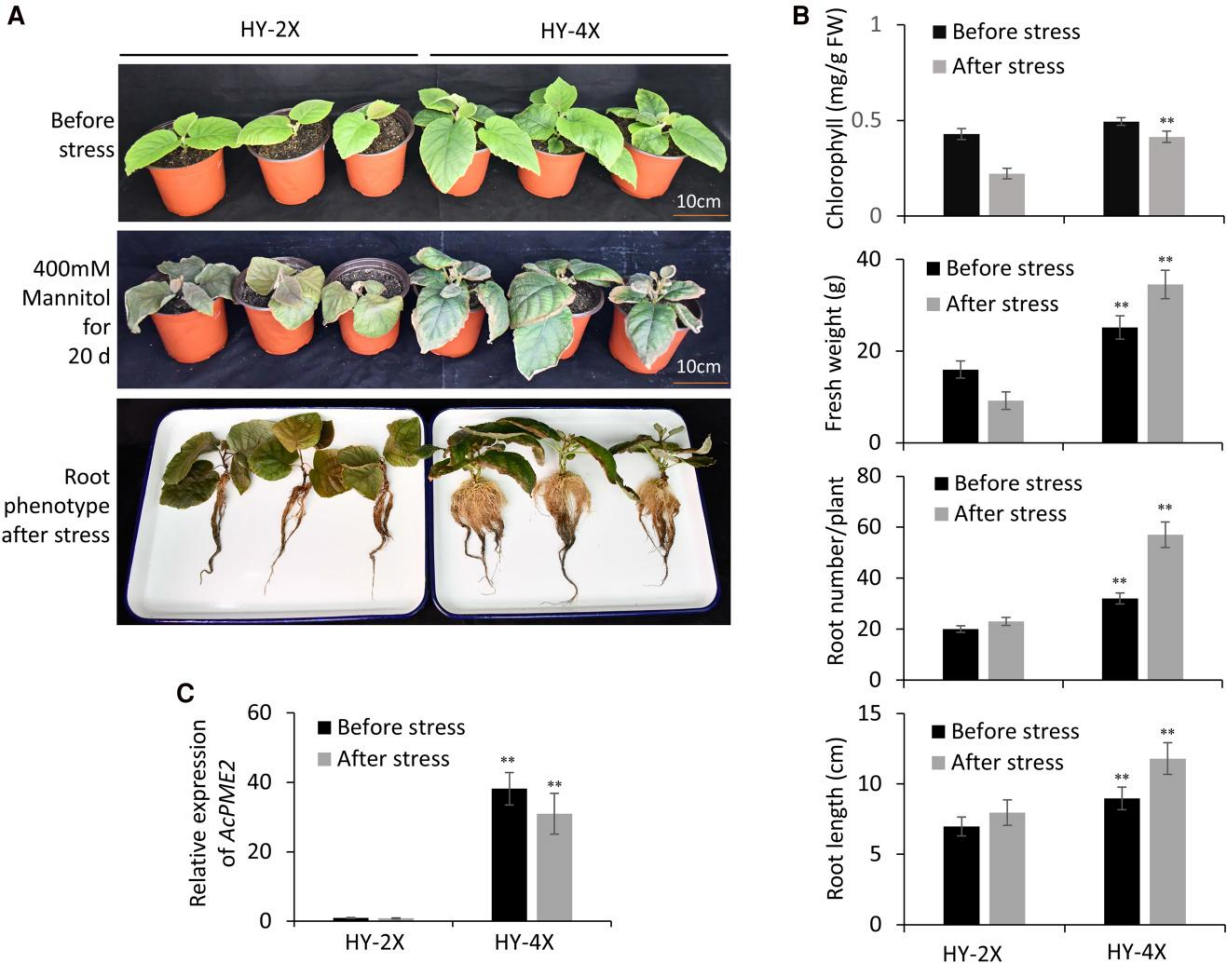
The tetraploid “Hongyang” is more tolerant to osmotic stress than the diploid one. **A)** The phenotypes of one-month-old “Hongyang (HY)” diploid (HY-2×) and tetraploid (HY-4×) plants (before stress) were watered with 400 mm mannitol for about 20 days (after stress). **B)** The fresh weight, chlorophyll content, root number/plant, and root length of the HY-2× and HY-4× plants before and after osmotic stress treatment. Mean ± SD were obtained from three independent experiments (n = 12). Significance was calculated by comparing the columns with the same color between HY-2× and HY-4× plants and indicated by **P < 0.001 (Student's *t*-test). **C)** The RT-qPCR analysis of AcPME2 gene expression in the HY-2× and HY-4× plants before and after osmotic stress treatment. Mean ± SD were obtained from three biological replicates (*n* = 3). Significance was calculated by comparing the columns with the same color between HY-2× and HY-4× plants and indicated by **P < 0.001 (Student's *t*-test).

We created the tetraploid (4×) “Hongyang” (HY) by treating diploid (2×) HY with colchicine in our previous study (Zhu et al. 2021). The 2-month-old plants of 2× and 4× HY were generated from tissue culture. The 4× plants have more biomass than 2× plants (Fig. 1A), as indicated by increased fresh weight, root length, and root number (Fig. 1B). Both 2× and 4× plants were treated with osmotic stress, as simulated by continuously watering with 400 mm mannitol for 20 days (Fig. 1A). After treatment, 2× plants showed wilting leaves while 4× plants remained stretched (Fig. 1A). The fresh weight, chlorophyll content, root number and length of 4× plants were significantly higher than that of 2× plants (Fig. 1B), suggesting that the tolerance to osmotic stress was also increased in 4× plants.

The transcriptomic analysis from our previous study indicated that several *PME* genes were significantly upregulated in the 4× plants (Zhu et al. 2021). One of *PMEs*, termed *AcPME2* (Actinidia06151), was remarkably increased in the 4× plants without or with the osmotic stress, as indicated by the RT-qPCR assays (Fig. 1C).

### Overexpression of *AcPME2* increased both biomass and stress tolerance in Arabidopsis, tomato, and kiwifruit

**Figure 2.**
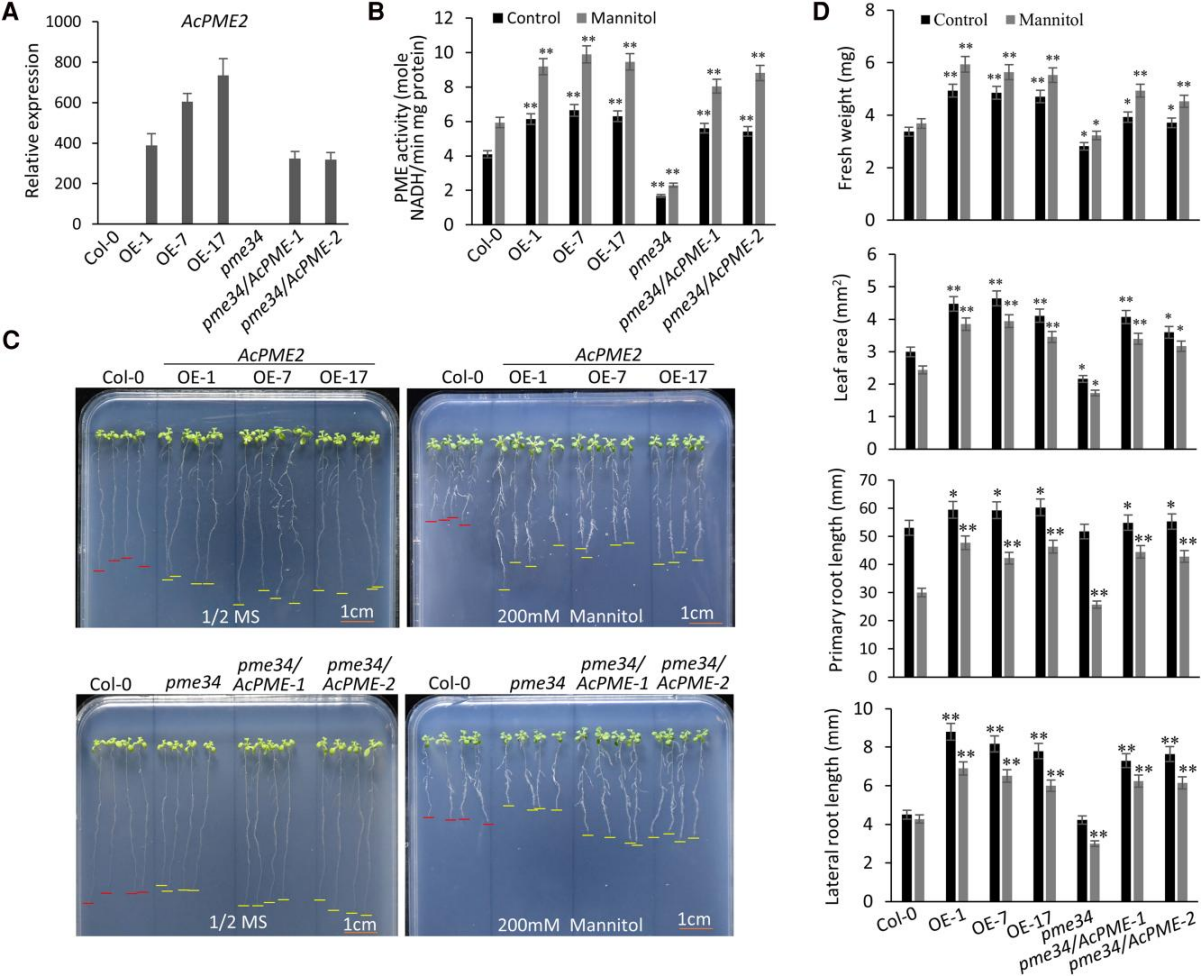
Overexpression of *AcPME2* increased the biomass and tolerance to osmotic stress in Arabidopsis. **A)** The RT-qPCR analysis of *AcPME2* gene expression in Arabidopsis Col-0, AcPME2-overexpressing (-OE) lines (-1, -7, and -17), *pme34* mutant, and complementary lines (*pme34/AcPME2-1* and -*2*). Mean ± SD were obtained from three biological replicates (*n* = 3). **B-D)** The PME activities of whole plants **(B)**, phenotypes **(C)**, and fresh weight, leaf area, primary root length, and lateral root length **(D)** of 6-day-old seedlings of Col-0, AcPME2-OE lines, *pme34* mutant, and complementary lines were cultured in the 1/2 mS media (Control) or 1/2 mS containing 200 mm mannitol for 1 week. Red and yellow lines marked the root tips of Col-0 and other lines, respectively. Mean ± SD were obtained from three independent experiments (*n* = 15). The significant difference was calculated by comparing the columns with the same color between Col-0 and other lines and indicated by “**” (*P* < 0.001, Student's *t*-test).

AcPME2 shows the high similarity of protein sequence to PME34 in Arabidopsis (Huang et al. 2017) and belongs to the type I PMEs, as indicated by the presence of PMEI and PME regions (Supplementary Fig. S1). We transformed Arabidopsis (Col-0) with *AcPME2* and obtained three overexpression lines (AcPME2-OE-1, -7, and -17), as confirmed by RT-qPCR assays (Fig. 2A). As expected, the PME activities of three transgenic lines are significantly higher than that of Col-0 (Fig. 2B). We observed that the three AcPME2-OE lines showed the increased biomass, as indicated by the increased fresh weight, leaf area, primary, and lateral root length (Fig. 2, C and D). The cell sizes of guard cell and epidermal cell were significantly increased in the AcPME2-OE lines (Supplementary Fig. S2). Meanwhile, the fresh weight, leaf area, primary, and lateral root length of three lines was remarkably higher under MS/2 media with 200 mm mannitol than that of Col-0 plants (Fig. 2, C and D), suggesting that *AcPME2* heterologous expression enhanced the tolerance to osmotic stress in Arabidopsis. The mutant *pme34* (Huang et al. 2017) showed the lower PME activities (Fig. 2B) and was sensitive to osmotic stress (Fig. 2, C and D). Overexpression of *AcPME2* could rescue the impaired PME activities and stress sensitivity of *pme34* (Fig. 2, C and D). It is worth noting that osmotic stress enhanced PME activities in both Col-0 and transgenic plants (Fig. 2B).

**Figure 3.**
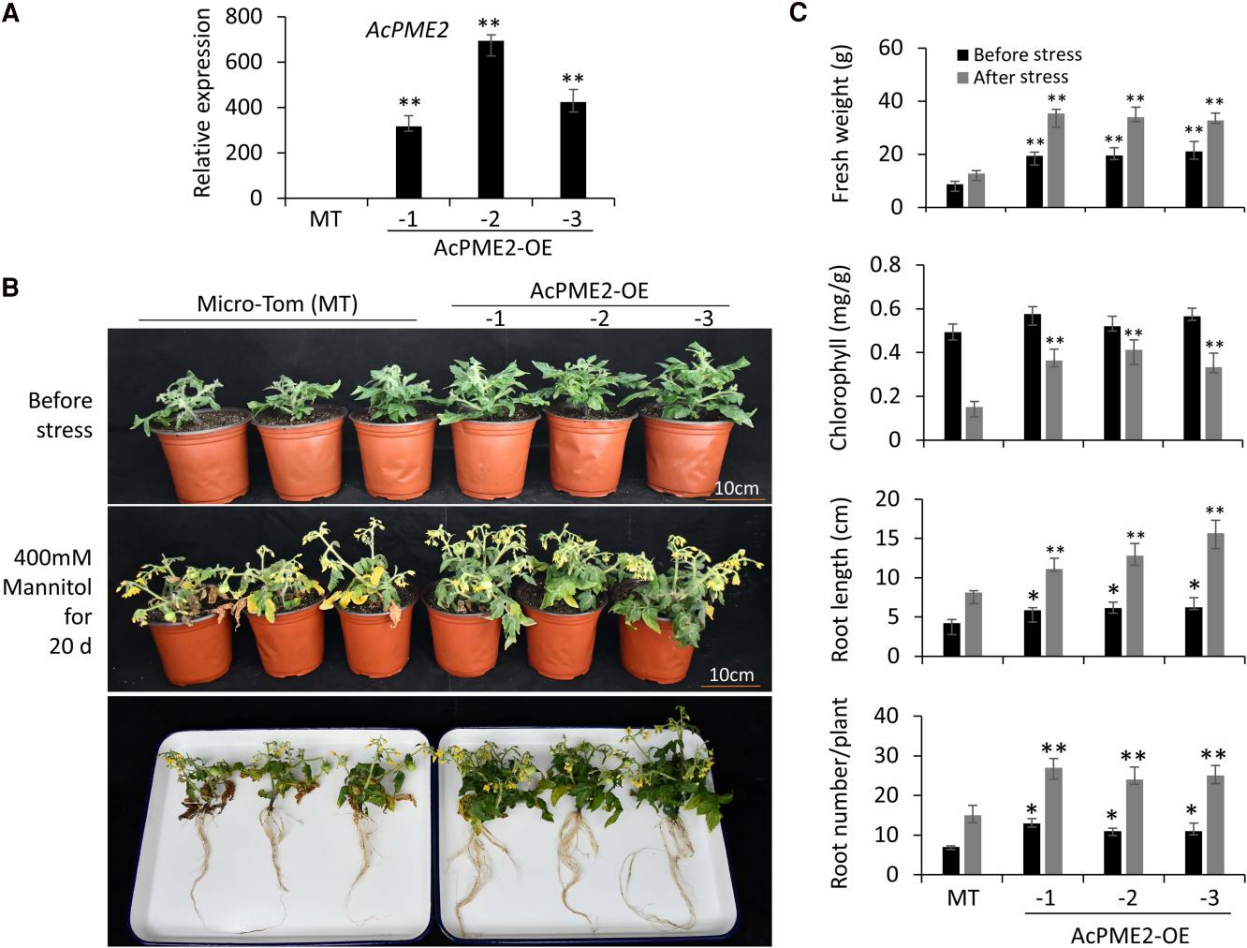
Overexpression of *AcPME2* increased the biomass and tolerance to osmotic stress in tomato. **A)** The RT-qPCR assays of *AcPME2* expression in the wild-type tomato micro-Tom (MT) and the transgenic AcPME2-overexpressing (-OE) lines. Mean ± SD were obtained from three biological replicates (*n* = 3). “**” indicates the significant difference (*P* < 0.001). **B and C)** The phenotypes (B), fresh weight, chlorophyll contents, root length and root number per plant **(C)** of MT and AcPME2-OE lines (before stress) were watered by 400 mm mannitol for about 20 days (after stress). Mean ± SD were obtained from three independent experiments (*n* = 15). The significant difference was calculated by comparing the columns with the same color between wild-type and transgenic lines and indicated by “**” (*P* < 0.001) and “*” (*P* < 0.05, Student's *t*-test).

We also obtained three heterologous expression tomato lines (AcPME2-OE-1, -2, and -3) by transforming Micro-Tom (MT) (Fig. 3A). As expected, the three transgenic lines showed the increased biomass (Fig. 3B), as indicated by the increased fresh weight, root length, and root number per plant (Fig. 3C). Similarly, the three lines showed the enhanced tolerance to osmotic stress (Fig. 3, B and C). Besides, the fruits of the three lines were larger than that of MT (Supplementary Fig. S3A), as indicated by the increased fresh weight of tomato fruits (Supplementary Fig. S3B). The transverse diameter of fruit, but not vertical diameter, was increased in the transgenic fruits (Supplementary Fig. S3B). These results indicated that *AcPME2* heterologous expression can increase the fruit size in tomato.

**Figure 4.**
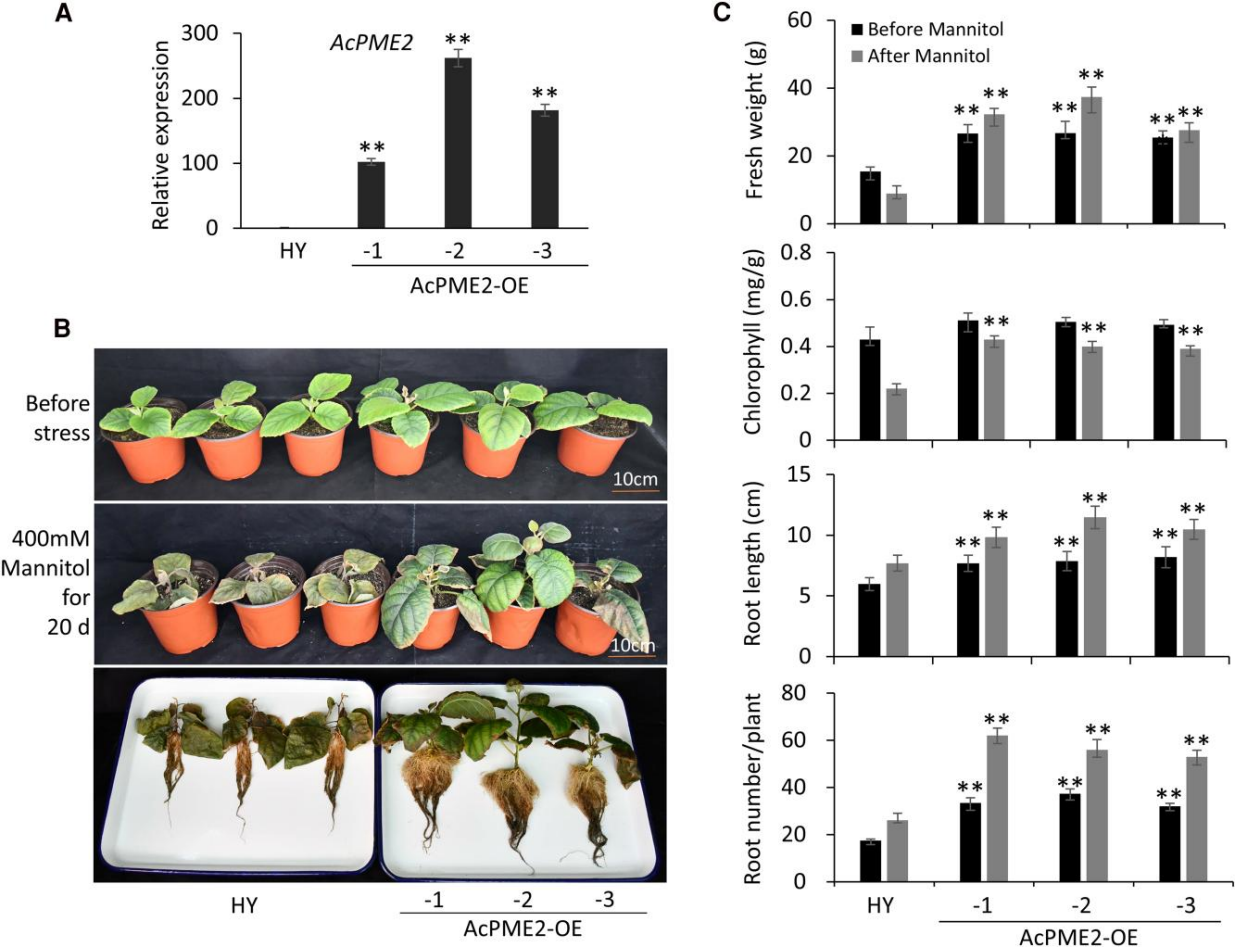
Overexpression of *AcPME2* increased the biomass and tolerance to osmotic stress in kiwifruit. **A)** The RT-qPCR assays of *AcPME2* expression in diploid “Hongyang”(HY) plants and the AcPME2-overexpressing (-OE) lines. Mean ± SD were obtained from three biological replicates (*n* = 3). “**” indicates the significant difference (*P* < 0.001, student's t-test). **B and C)** The phenotypes **(B)**, fresh weight, chlorophyll contents, root length and root number per plant **(C)** of HY and the AcPME2-OE lines (before stress) were watered by 400 mm mannitol for about 20 days (after stress). Mean ± SD were obtained from three independent experiments (*n* = 12). The significant difference was calculated by comparing the columns with the same color between wild-type and transgenic lines and indicated by “**” (*P* < 0.001, Student's *t*-test).

We also created three transgenic lines (AcPME2-OE-1, -2, and -3) of *Actinidia Chinensis* “Hongyang” (HY) by agrobacterium-mediated transformation and *AcPME2* expression was confirmed by RT-qPCR (Fig. 4A). Before stress, the transgenic plants showed the increased fresh weight, root length, and root number per plants (Fig. 4, B and C), suggesting that *AcPME2* overexpression can increase the biomass in kiwifruit. After osmotic stress treatment, the leaves of HY wild type plants were wilting but those of AcPME2-OE lines were not (Fig. 4B). The fresh weight of HY plants was decreased but that of three transgenic lines were remarkably increased (Fig. 4C). The chlorophyll content, root length, and root number of transgenic lines were much higher than that of HY plants after stress treatment (Fig. 4C). These results indicated that *AcPME2* overexpression increased both biomass and stress tolerance in kiwifruit.

### AcPME2 promotes pectin demethylesterification and calcium accumulation in cell wall in the early stage of stress response

**Figure 5.**
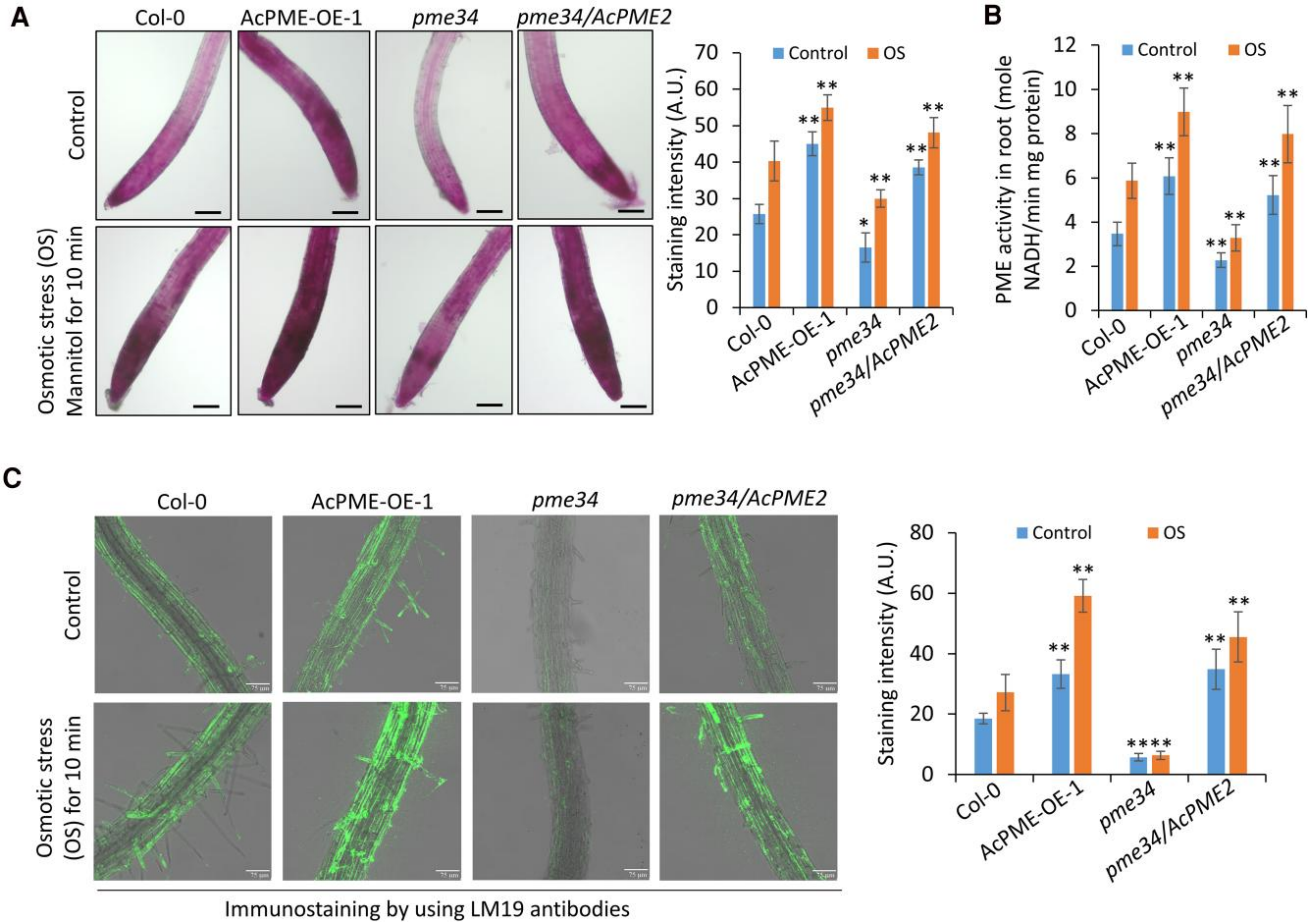
Osmotic stress enhanced pectin demethylesterification in Arabidopsis. **A and B)** The ruthenium red staining **(A)** and PME activities **(B)** of roots of Col-0, AcPME2-OE-1, *pme34* mutant, and complementary line *pme34/AcPME2-1* treated in liquid MS/2 media without (control) or with 200 mm mannitol for 10 min (osmotic stress, OS). Bar = 100 *μ*m. **C)** Immunostaining of roots of above-mentioned lines treated with 200 mm mannitol for 10 min by using LM19 antibodies recognizing the demethylesterified pectin. Bar = 75 *μ*m. The staining intensity was quantitatively measured using ImageJ. Mean ± SD were obtained from three biological replicates (*n* = 3). The significant difference was calculated by comparing the columns with the same color between Col-0 and other lines. “*” and “**” indicate the significant difference at *P* < 0.05 and *P* < 0.001 (Student's *t*-test), respectively.

To visualize the demethylesterified pectin, roots of Col-0 and transgenic Arabidopsis plants were stained with 0.01% ruthenium red, according to the methods described in the previous studies (Asahina et al. 2002; Wu et al. 2010). As compared with roots of Col-0, the AcPME2-OE roots showed higher density of red color, while the mutant *pme34* showed lower density staining (Fig. 5A), suggesting that AcPME2-OE roots had more demethylesterified pectins but *pme34* had less. The immunostaining with LM19 antibodies, which recognizes the demethylesterified pectin, also showed the same results (Fig. 5C). These observations are consistent with the above-mentioned results that the transgenic plants had higher but *pme34* mutant had lower PME activities (Fig. 2B). Meanwhile, we observed that the short-term treatment of osmotic stress (200 mm mannitol for 10 min) enhanced the pectin demethylesterification (Fig. 5, A and C) and PME activities in roots (Fig. 5B). After the stress treatment, the AcPME2-OE roots showed higher levels but *pme34* roots showed lower levels of pectin demethylesterification and PME activities than Col-0 roots (Fig. 5), suggesting that AcPME2 promotes pectin demethylesterification in the early stage of osmotic stress response. AcPME2-enhanced demethylesterification of pectin was also observed in the kiwifruit roots (Supplementary Fig. S4, A and B).

**Figure 6.**
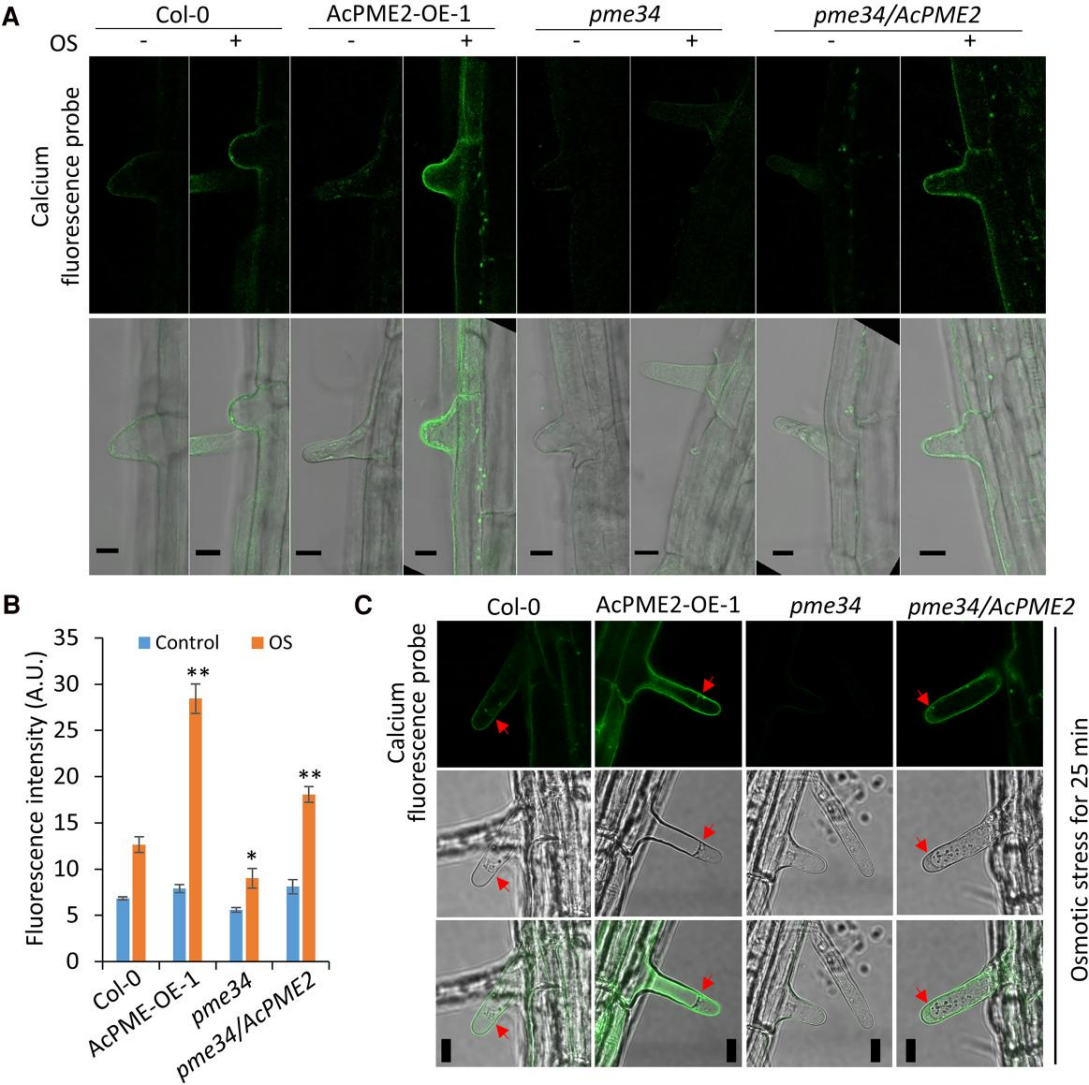
The osmotic stress induced calcium accumulation in cell wall in Arabidopsis. **A and B)** The confocal microscope observation **(A)** and fluorescence intensity **(B)** of calcium fluorescence probe (fluo-4/AM) in the roots of above-mentioned lines treated without (control, −) or with 200 mm mannitol for 10 min (OS, +). The fluorescence intensity was quantitatively measured by using ImageJ. Mean ± SD were obtained from three biological replicates (*n* = 3). The significant difference is calculated by comparing the columns with the same color between Col-0 and other lines and indicated by “**” (*P* < 0.001) and “*” (*P* < 0.05, Student's *t*-test). **C)** The confocal microscope observation of calcium fluorescence probe (fluo-4/AM) in the roots of above-mentioned lines treated with 200 mm mannitol for 25 min. Bar = 20 *μ*m. Red arrows indicate the plasmolysis in root hair.

Meanwhile, we used calcium fluorescence probe (fluo-4/AM) to detect Ca^2+^ in the roots. The confocal microscope observations indicated that, under control condition, there is no obvious difference on Ca^2+^ concentrations between Col-0 and AcPME2-OE roots (Fig. 6, A and B). However, after the short-term treatment of osmotic stress, the cell wall of AcPME2-OE roots accumulated more Ca^2+^ than that of Col-0 roots (Fig. 6, A and B). Stress-induced accumulation of Ca^2+^ on cell wall was impaired in *pme34* mutant but *AcPME2* overexpression in the mutant could rescue the defect (Fig. 6, A and B), suggesting AcPME2 can enhance the calcium accumulation on the cell wall. To determine whether Ca^2+^ was really accumulated in cell wall, we extended the treatment to induce the plasmolysis of root hairs (as indicated by the red arrows in Fig. 6C). The fluorescence probe of Ca^2+^ was still associated with the cell wall of root hairs when the plasmolysis occurred (Fig. 6C), suggesting that Ca^2+^ really bound to the cell wall. The accumulation of more Ca^2+^ was also observed on the cell walls of transgenic kiwifruit roots (Supplementary Fig. S4C), suggesting that PME-enhanced Ca^2+^ accumulation under osmotic stress might be conserved in the kiwifruit.

### AcPME2 affects osmotic stress-induced plasmolysis in root hair

**Figure 7.**
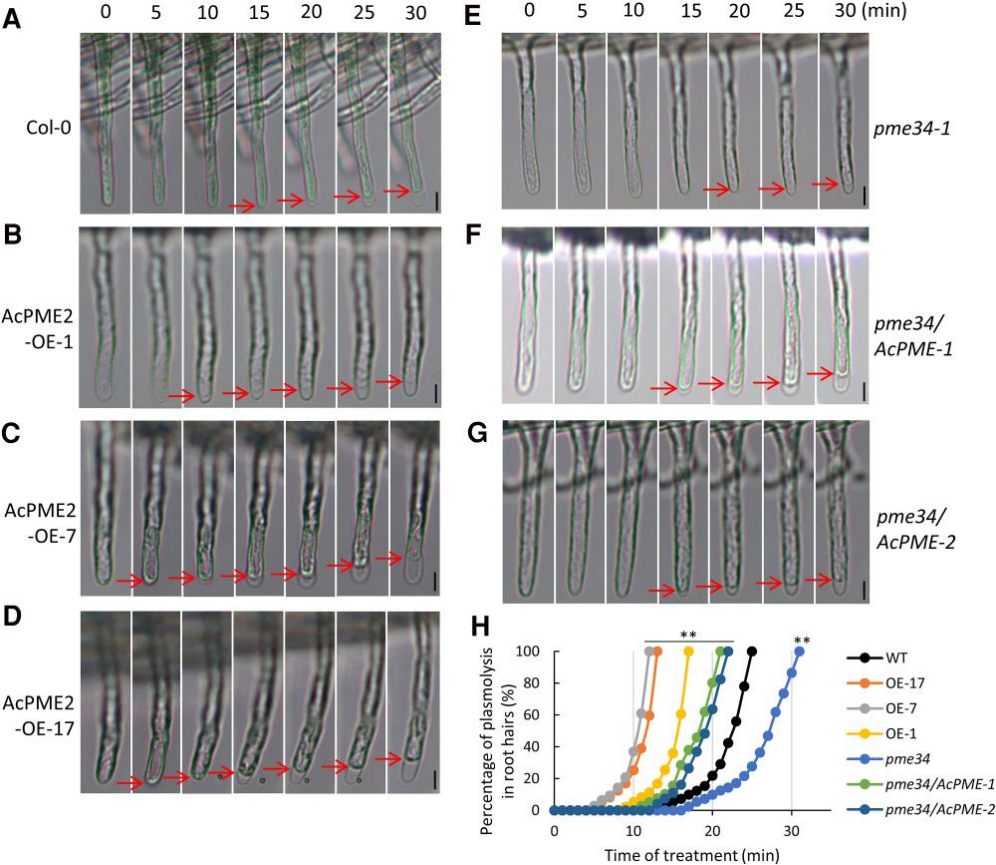
AcPME2 affects osmotic stress-induced plasmolysis in root hair in Arabidopsis. **A-G)** Microscope observation of plasmolysis in the root hairs of Col-0 **(A)**, AcPME2-OE-1 **(B)**, AcPME2-OE-7 **(C)**, AcPME2-OE-17 **(D)**, *pme34***(E)**, *pme34/AcPME-1***(F)**, and *pme34/AcPME-2***(G)** treated in the liquid MS/2 media containing 200 mm mannitol for 30 min. Bar = 10 *μ*m. **H)** Percentage of plasmolysis in the root hairs of above-mentioned lines with the same treatment. For each line, more than 20 root hairs from three independent experiments (*n* = 20) were observed and accounted for the percentage. The significant difference is calculated by comparing wild type with other lines and indicated by “**” (*P* < 0.001, Student's *t*-test).

The free carboxyl groups of demethylesterified pectin could interact with Ca^2+^ to form a pectate gel that results in cell wall stiffening (Micheli 2001). Our results showed that osmotic stress caused more demethylesterified pectins and more Ca^2+^ accumulation in the cell wall, which might result in the cell wall stiffening. Therefore, we speculate that the cell wall of AcPME2-OE plants might be stiffer than that of Col-0 under stress. The stiffer cell wall causes higher tension between cell wall and protoplasm under osmotic stress, which might result in faster plasmolysis. Therefore, we compared the speed of osmotic stress-induced plasmolysis in root hairs of Col-0 and transgenic lines. The young seedlings of Col-0 and transgenic lines were immerged into liquid MS/2 media with 200 mm mannitol and root hairs were continuously observed by light microscope for 40 min. In Col-0, the plasmolysis of root hair was observed from 15 min (red arrows in Fig. 7A) and 100% of root hairs showed plasmolysis at about 25 min (Fig. 7H). In AcPME2-OE lines, the root hairs showed plasmolysis from 5 min or 10 min (red arrows in Fig. 7B-7D) and 100% showed plasmolysis at 11 min or 16 min (Fig. 7H). These results indicated that the plasmolysis of root hair in AcPME2-OE lines is faster than in Col-0, which might be caused by the stiffer cell walls of AcPME2-OE plants under osmotic stress. The root hairs of *pme34* mutant started to show plasmolysis from 20 min (Fig. 7E) and finished 100% at 30 min (Fig. 7H), which was slower than those of Col-0. The expression of *AcPME2* in *pme34* mutant could rescue the delayed plasmolysis in root hair under stress (Fig. 7F–H). Similarly, stress-induced plasmolysis was faster in tetraploidy kiwifruit plants and in AcPME2-OE plants than that in diploidy plants (Supplementary Fig. S5, A to D), suggesting that tetraploid and transgenic kiwifruit plants also showed stiffer cell wall under osmotic stress.

If the stress-induced stiffening of cell wall was caused by the accumulation of Ca^2+^, exclusion of Ca^2+^ should decrease the stiffness of cell wall, which might delay the plasmolysis. Indeed, we used the special MS/2 liquid media (containing no Ca^2+^) with 200 mm mannitol to do the same treatment and our results indicated that the stress-induced plasmolysis was significantly slower in both Col-0 and AcPME2-OE plants than what happened under treatment with Ca^2+^ (Supplementary Fig. S6). These results indicated that exclusion of Ca^2+^ might decrease the cell wall strength, which thereby delayed the plasmolysis in response to osmotic stress. The stress-induced plasmolysis of AcPME2-OE lines was even slower than that of Col-0 under the treatment without Ca^2+^ (Supplementary Fig. S6), suggesting that the cell wall of AcPME2-OE lines might be more flexible than that of Col-0 under control conditions (without Ca^2+^ accumulation). These results indicated that AcPME2 might dynamically mediate the cell wall stiffness, which is dependent on the alteration of Ca^2+^ concentrations in cell wall.

### *AcPME2* overexpression prevented cell wall collapse under osmotic stress in Arabidopsis and kiwifruit

**Figure 8.**
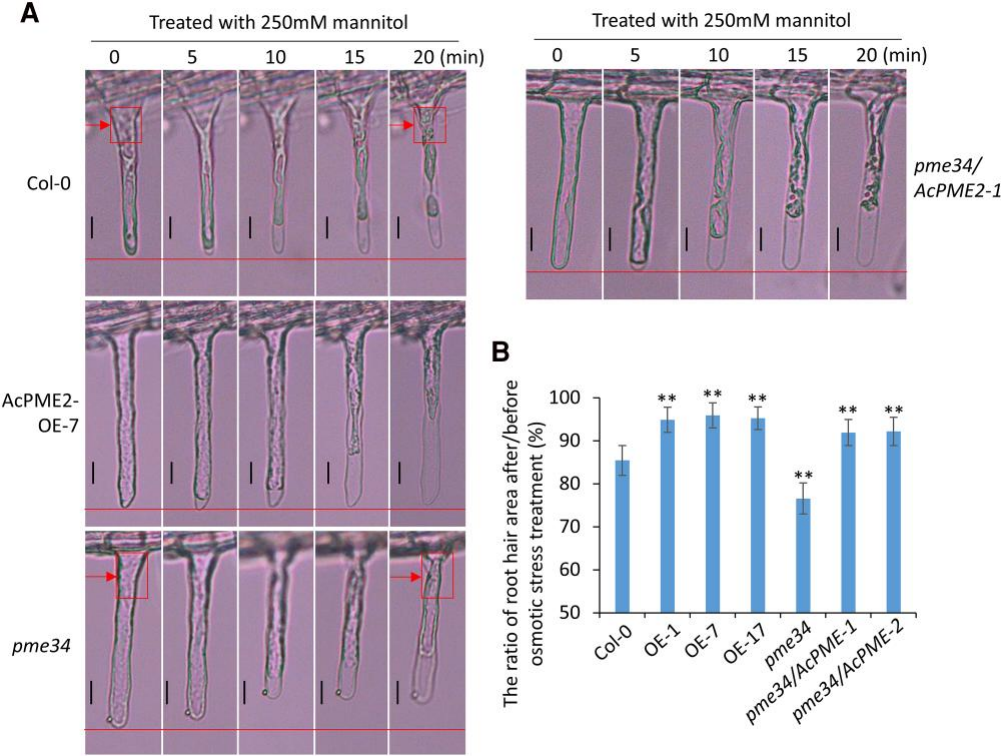
*AcPME2* prevented the cell wall collapse of root hairs under osmotic stress in Arabidopsis. **A)** Microscope observation of the root hairs of Col-0, AcPME2-OE-7, and *pme34* mutant treated in the liquid MS/2 media containing 250 mm mannitol for 30 min. Bar = 10 *μ*m. **B)** The ratio (Percentage) of root hair area after/before osmotic stress (250 mm mannitol) treatment for 30 min. For each line, mean ± SD were obtained from 5 root hairs from three independent experiments and their areas were measured by ImageJ. The significant difference is calculated by comparing Col-0 with other lines and indicated by “**” (*P* < 0.001, Student's *t*-test).

When we used higher concentrations of mannitol (250 mm) to perform the treatment, the cell wall collapse, and deformation, besides the plasmolysis, was observed in the Col-0, as indicated by the red arrows in Fig. 8A. The osmotic stress-induced cell wall collapse and deformation was even more severe in root hairs of *pme34* mutant and the obvious shrinkage of root hair was also observed in *pme34* (Fig. 8A). No obvious collapse of cell wall and shrinkage of root hair was observed in the AcPME2-OE and *pme34/AcPME2-OE* plants (Fig. 8A). The quantitative measurements of root hair areas after and before osmotic stress indicated that the root hair areas of Col-0 and *pme34* showed 15% and 25% reduction after stress treatment, respectively (Fig. 8B), while those of AcPME2-OE and *pme34/AcPME2-OE* plants showed less than 10% reduction (Fig. 8B). In kiwifruit, the osmotic stress-induced cell wall collapse and shrinkage was observed in root hair of 2× HY (as indicated by the magenta arrows in Supplementary Fig. S5C), while that is not obvious in the AcPME2-OE plants (Supplementary Fig. S5C). The root hair areas of AcPME2-OE plants showed less reduction after osmotic stress than those of 2× HY (Fig. 5E). These results indicated that *AcPME2* overexpression prevented the call wall collapse and shrinkage under osmotic stress, which reflected the increased cell wall stiffness of AcPME2-OE plants.

### *AcPME2* overexpression enhanced the expression of stress-responsive genes in the early stage of stress response

**Figure 9.**
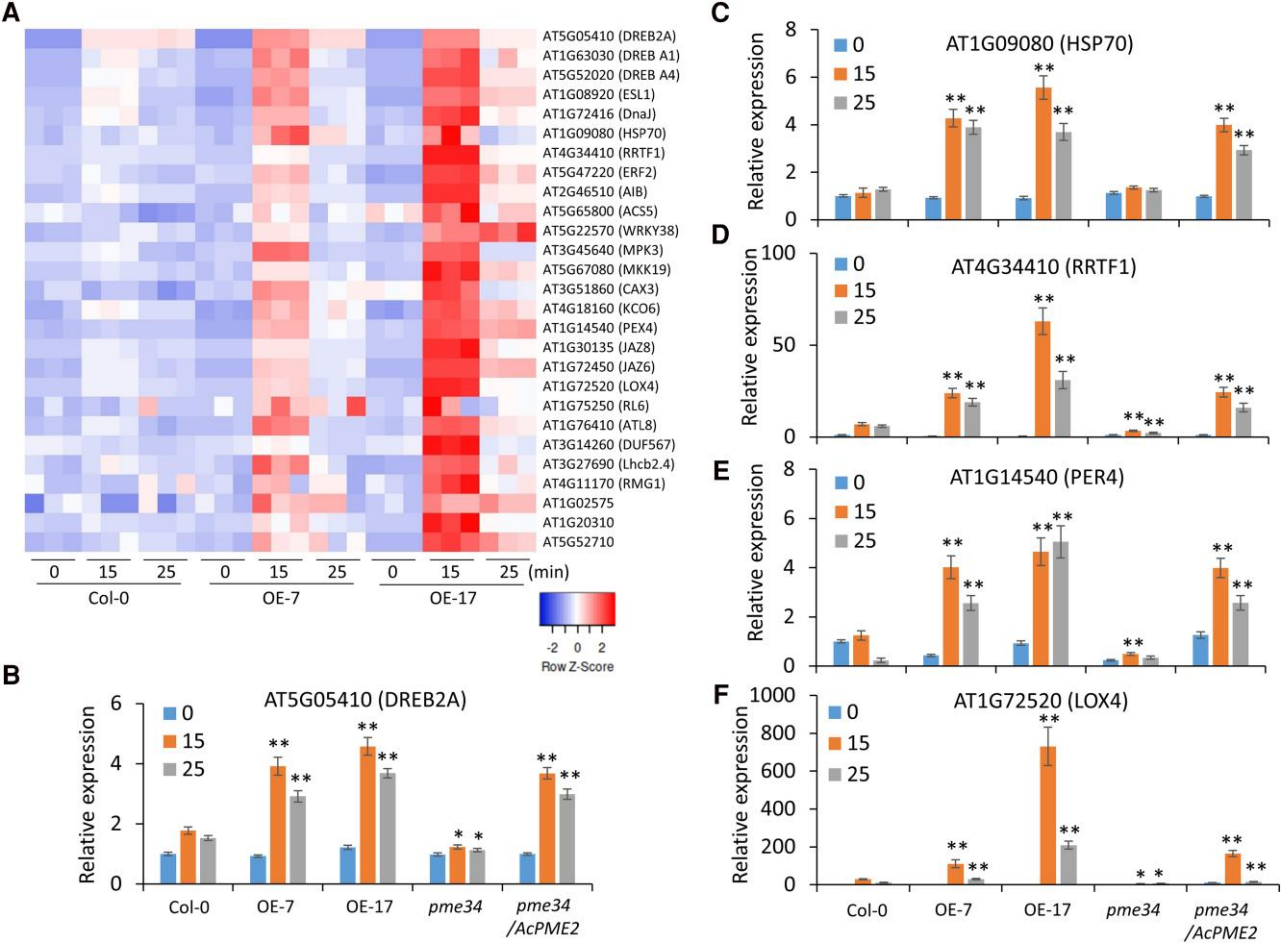
*AcPME2* overexpression upregulated stress-responsive genes in early response to osmotic stress. **A)** The differentially expressed stress-responsive genes identified from the transcriptomic analysis of Col-0, AcPME2-OE-7, and AcPME2-OE-17 roots treated in the liquid MS/2 media containing 200 mm mannitol for 0, 15, and 25 min. **B-F)** The RT-qPCR analysis of *DREB2A* (B), *HSP70* (C), *RRTF1* (D), *PER4* (E), and *LOX4* (F) expressions in the above-mentioned lines with the same treatment. Mean ± SD were obtained from three biological replicates (*n* = 3). The significant difference is calculated by comparing the columns with the same color between Col-0 and other lines and indicated by “**” (*P* < 0.001) and “*” (*P* < 0.05, Student's *t*-test).

The abiotic stress causes the mechanical distortion of cell wall and changes the physical forces at the cell wall-plasma membrane interphase, which serves as a signal to mediate the expression of downstream genes (Vaahtera et al. 2019; Gigli-Bisceglia et al. 2022). Therefore, we performed the transcriptomic analysis of roots of Col-0 (WT) and AcPME2-OE lines (-7 and -17) under osmotic stress treatment for 0, 15, and 25 min. The principal component analysis (PCA) of total 27 samples showed the high repeatability and consistency of these experimental datasets (Supplementary Fig. S7A). Totally, 5,517 differentially expressed genes (DEGs) were identified (Table SI). Among them, 28 DEGs were activated by osmotic stress and showed higher expression in AcPME2-OE lines than in Col-0 under stress (Supplementary Fig. S7B and Table SII). These DEGs included stress-responsive transcription factors (DEHYDRATION-RESPONSIVE ELEMENT BINDING PROTEINs, DREBs), molecular chaperone, MAP kinase, calcium transporter, Jasmonic acid (JA) pathway, and so on (Fig. 9A). The RT-qPCR analysis confirmed that the mRNA levels of DREB2A, HEAT SHOCK PROTEIN 70 (HSP70), REDOX RESPONSIVE TRANSCRIPTION FACTOR 1 (RRTF1), peroxidase 4 (PER4), and LIPOXYGENASE 4 (LOX4) in AcPME2-OE lines were significantly higher than in Col-0 under stress (Fig. 9, B to F), which were consistent with the transcriptomic data (Fig. 9A). The results indicated that overexpression of *AcPME2* enhanced the expression of stress-responsive genes in the very short-term treatment of osmotic stress. Meanwhile, we also confirmed that the stress-mediated activations of these genes were impaired in *pme34* but rescued in the complementary lines (Fig. 9, B to F).

## Discussion

Chromosome doubling-induced polyploidization is a powerful tool and has been extensively applied in crop breeding. Polyploidization commonly results in the increased biomass yield, the enhanced stress tolerance, and the improved quality traits. The most common consequence of polyploidy in plants is the increased cell size, which is termed as the “gigas” effect and leads to larger organs and increased biomass (del Pozo and Ramirez-Parra 2015; Sattler et al. 2016). However, the gene mediating the “gigas” effect is largely unknown. Our previous study indicated that colchicine-induced chromosome doubling in kiwifruit also led to the increased cell size and biomass (Zhu et al. 2021). And our transcriptomic analysis revealed that several *PME* genes were upregulated in the tetraploid “Hongyang”. In this study, we successfully overexpressed *AcPME2* in Arabidopsis, tomato, and kiwifruit and all the transgenic plants displayed the increased biomass, as indicated by the increased fresh weight, leaf area, and root length (Figs. 2 to 4). In tomato, *AcPME2* overexpression also increase the fruit size (Supplementary Fig. S3). These results indicated that chromosome doubling-induced high expression of *AcPME2* contributed to the increased biomass in the kiwifruit. The previous studies indicated that, when acting in a random way, PME-mediated demethylesterification of pectins makes homogalacturonans (HGs) available for HG-modifying enzymes, such as pectate lyases (Colin et al. 2023), or provides the platforms that anchor peroxidases to remodel plant cell wall for cell wall loosening and cell extension (Moustacas et al. 1991; Francoz et al. 2019). Our results indicated that AcPME2-OE plants showed higher levels of pectin demethylesterification (Fig. 5A), which might lead to the cell wall loosening, as indicated by the increased cell size (Supplementary Fig. S2). Our results indicated that *AcPME2* is one of genes responsible for the “gigas” effect from polyploidization.

Meanwhile, the tetraploid “Hongyang” plants showed the better tolerance to osmotic stress than the diploid one (Fig. 1). The overexpression of *AcPME2* also enhanced the tolerance to osmotic stress in Arabidopsis (Fig. 2), tomato (Fig. 3), and kiwifruit (Fig. 4). As compared with control plants, stress-tolerant crop plants usually showed impaired growth and development, which leads to the yield penalty (Brown 2002; Mickelbart et al. 2015). However, our results indicated that the overexpression of *AcPME2* contributed to both increased biomass and enhanced tolerance to osmotic stress in different plants. It is quite intriguing that these two advantages were achieved by manipulating a single gene involved in cell wall modification.

Cell wall not only plays a key role in plant growth and development, but also participates in the perception of environmental stresses and stress response (Anderson and Kieber 2020). Cell wall integrity (CWI) is impaired when plants are subjected to biotic or abiotic stress. Monitoring CWI involves the perception of the physical forces at cell wall-plasma membrane interphase (Vaahtera et al. 2019). Abiotic stress such as drought, salinity, and osmotic stress, causes the protoplast shrinkage and the mechanical distortion between cell wall and plasma membrane, due to reduced cellular turgor pressure resulted from the water escaping (Zhu 2016; Vaahtera et al. 2019; Colin et al. 2023). The mechanical distortion can be perceived by the sensors, such as wall-associated kinases (WAKs) and receptor-like kinases. For example, the receptor-like kinase THE1 is responsible for sensing mechanical stress of cell wall upon cellulose impairment (Hematy et al. 2007). Mid1-Complementing Activity 1 (MCA1) is a mechanosensitive Ca^2+^ channel that is activated by plasma membrane distortion (Nakagawa et al. 2007) and functions downstream of THE1 in CWI perception (Engelsdorf et al. 2018). Pectin also plays an indispensable role in call wall perception by direct interaction with these sensors. The receptor-like kinase FER interacts with pectin to sense salinity-induced cell wall defects and maintains CWI through Ca^2+^ signaling (Feng et al. 2018). Besides, the PME-mediated de-esterification of pectin is required for WAK activation in the pathogen response (Kohorn et al. 2014) and salt stress response (Gigli-Bisceglia et al. 2022). Although these results revealed the involvement of pectin in stress perception and response, the underlying mechanisms are poorly understood.

Our results indicated that the osmotic stress treatment rapidly enhanced the PME activities and Ca^2+^ accumulation in the cell wall of Col-0 roots, in 10 min (Fig. 5). We speculate that Ca^2+^ accumulation might lead to the cell wall stiffening. This is because, when acting in a linear way, PME produces continuous blocks of free carboxyl groups that could interact with Ca^2+^ to form a pectate gel (Micheli 2001; Willats et al. 2001). In AcPME2-OE plants, the levels of de-esterified pectin and Ca^2+^ accumulation in the cell wall were higher than in Col-0 plants (Figs. 5 and 6), suggesting that the cell wall of AcPME2-OE plants might be stiffer than that of Col-0 plants under osmotic stress. Plasmolysis, the dissociation of protoplasm from the cell wall, is caused by the shrinkage of protoplasm due to dehydration. If the cell wall is soft and flexible, it will delay the plasmolysis because the flexible cell wall would curve inward, along with the shrinkage of protoplasm. If the cell wall is stiff and hard to curve, it will facilitate the plasmolysis. Our results indicated that the osmotic stress-induced plasmolysis was faster in AcPME2-OE plants than in Col-0 (Fig. 7), which are consistent with our speculation that the cell wall of AcPME2-OE was stiffer than that of Col-0. Stress-induced pectin de-esterification and Ca^2+^ accumulation was impaired in *pme34* mutant but rescued by overexpression of *AcPME2* in the mutant (Fig. 5). Accordingly, stress-induced plasmolysis was delayed in *pme34* mutant, which could be rescue by *AcPME2* overexpression (Fig. 7). Our results indicated that *AcPME2* increases the stiffness of cell wall under osmotic stress, via affecting Ca^2+^ accumulation in the cell wall, and thereby promotes stress-induced plasmolysis. AcPME2-induced faster plasmolysis under osmotic stress occurs not only in Arabidopsis, but also in kiwifruit (Supplementary Fig. S5). Under higher concentrations of mannitol, the cell wall collapse and deformation were clearly observed in the root hairs of wild type plants and *pme34* mutant, but not in those of AcPME2-OE plants in Arabidopsis (Fig. 8) and in kiwifruit (Supplementary Fig. S5, C and E). These results also indicated that *AcPME2* overexpression increased the cell wall stiffness.

The Ca^2+^ is key player in the stress-induced cell wall stiffening, because Ca^2+^ exclusion significantly delayed the plasmolysis in both Col-0 and AcPME2-OE plants (Supplementary Fig. S6). Interestingly, the plasmolysis of AcPME2-OE roots was even slower than that of Col-0 in the absence of Ca^2+^ (Supplementary Fig. S6), suggesting that the cell walls of AcPME2-OE lines might be softer or more flexible than that of Col-0 under control condition (without Ca^2+^ accumulation). These results indicated that AcPME2 could dynamically mediate the cell wall stiffness according to stress-induced alteration of Ca^2+^ concentrations in cell wall. Besides, the higher flexibility of AcPME2-OE cell walls in absence of Ca^2+^ accumulation is consistent with the previous studies indicating that PME-mediated demethylesterification causes cell wall loosening (Moustacas et al. 1991; Francoz et al. 2019), which contributed to the increased biomass of AcPME2-OE plants under control condition (Figs. 2 to 4).

As compared with Col-0 plants, the increased stiffness of cell wall in AcPME2-OE plants under osmotic stress should cause the earlier and higher tension and more server mechanical distortion between cell wall and plasma membrane, as indicated by the faster plasmolysis in AcPME2-OE plants (Fig. 7). The earlier tension and more server mechanical distortion might be perceived more rapidly in the AcPME2-OE plants by the above-mentioned sensors. The perceived signal in AcPME2-OE plants, which should be stronger than in Col-0, can transduce to the downstream stress-responsive genes and lead to the upregulation of their expression. Indeed, our transcriptomic analysis revealed that dozens of stress-responsive genes were activated by osmotic stress and showed higher expression in AcPME2-OE lines than in Col-0 under stress (Fig. 9). These genes encode stress-responsive transcription factors, molecular chaperone, MAP kinases, calcium transporter, JA pathway, and so on (Fig. 9). Drought-induced transcription factor DREB2A significantly enhanced water stress tolerance by binding to the dehydration-responsive elements of stress-inducible genes (Sakuma et al. 2006). DNaJ and HSP70 are molecular chaperones preventing protein misfolding under stress. RRTF1 is a key regulator maintaining redox homeostasis when plants were subjected to abiotic stress (Khandelwal et al. 2008). *LOX4* encodes a principal enzyme responsible for wound-induced production of JA precursors (Yang et al. 2020) and JA plays a positive role in response to abiotic stress (Kazan 2015). The higher expression of these genes under osmotic stress can contribute to the enhanced stress tolerance in AcPME2-OE plants. Interestingly, *RRTF1* expression was also altered in the *fer-4* mutant and *herk1 the1-4* double mutant in response to salt stress (Gigli-Bisceglia et al. 2022), suggesting that the sensors FER, HERK1, and THE1 might be involved in the AcPME2-mediated regulation of stress-responsive genes.

In addition to the role in the signal perception, the increased cell wall stiffness caused by *AcPME2* overexpression might also provide the stronger mechanical support for plants subjected to osmotic stress. The mechanical strength of cell wall is important for plant development and stress response. Defects on some genes of cell wall biosynthesis caused the hypersensitivity to salt and osmotic stress (Colin et al. 2023; Dabravolski and Isayenkov 2023). Overexpression of *UGE3* (*UDP-galactose/glucose epimerase 3*) from rice (*Oryza sativa*) enhanced biosynthesis of cellulose and hemicellulose, which led to increased mechanical strength of cell wall and thereby enhanced the tolerance to osmotic and salt stress (Tang et al. 2022). In our study, osmotic stress-induced cell wall collapse and deformation was observed in the Col-0 and *pme34* mutant (Fig. 8). The severe cell wall collapse and shrinkage in *pme34* mutant (Fig. 8) indicated the severe damage of cell wall, which might also contribute to the hypersensitivity to osmotic stress. *AcPME2* overexpression obviously prevented the cell wall collapse and shrinkage in root hairs of Arabidopsis and kiwifruit (Fig. 8 and Supplementary Fig. S5) by increasing the cell wall stiffness. Besides, the leaves of wild type tomato or kiwifruit plants were wilting after osmotic stress treatment but that of AcPME2-OE plants maintained stretched (Figs. 3B and 4B). These results indicated that AcPME2-OE plants had the stiffer cell wall than Col-0 and *pme34* mutant, which could provide the mechanical protection and prevent mechanical damages from osmotic stress.

Based on our results, we speculate the working mode of AcPME2. Under control condition, AcPME2 works in a random way to promote cell wall loosening and expansion, which contributes to the increased biomass. However, osmotic stress increases AcPME2 activities and might also change its working mode from a random way to a linear way. AcPME2 produced more continuous blocks of free carboxyl groups for Ca^2+^ binding, which increased the stiffness of cell wall. The stiffer cell wall produces the stronger signal of mechanical distortion, which thereby increases the expressions of stress-responsive genes and stress tolerance. Of course, it requires further investigations to determine how osmotic stress alters the working mode of AcPME2 and how AcPME2 affects stress-induced Ca^2+^ accumulation in cell wall under.

Together, our results revealed a single gene responsible for two advantages from polyploidization (the increased biomass and stress tolerance). We also discover a previously unknown mechanism that *AcPME2* dynamically regulates cell wall stiffness in response to osmotic stress. Besides, our work identified a cell-wall localized protein mediating the osmotic stress-induced plasmolysis and cell wall collapse, which further extended our knowledge about cell wall's role in stress response.

## Materials and methods

### Plant materials and the treatment of osmotic stress

The tetraploid *Actinidia chinensis* var. “Hongyang” were obtained from our previous study (Zhu et al. 2021). The explants of diploid and tetraploid “Hongyang” were harvested from the experimental base of Anhui Agricultural University. After sterilized, the explants were cultured on MS medium supplemented with 3 mg/L zeaxin (ZT) and 0.1 mg/L NAA under 25℃ for callus induction and shoot development. The generated shoots were transferred to the rooting media (MS/2 media plus 1 mg/L IBA). The generated plants were transferred into pots with the same amount of soils, cultured in the growth chamber (25℃, 16 h light/8 h dark) for 2 months and then subjected for the mannitol treatment.

The 2-month-old plants of diploid and tetraploid “Hongyang” generated from tissue culture were watered with 80 ml of 400 mm mannitol per three days. After 20 days, the treated plants were photographed and analysed. The transgenic diploid “Hongyang” plants were treated with osmotic stress in the same way.

### Vector construction and stable genetic transformation

The full-length cDNA sequence *AcPME2* (Actinidia06151) was cloned using RT-PCR and sequenced by Sanger sequencing. The coding region of *AcPME2* was inserted into the pHB (Li et al. 2016) by using HindIII and SacI, and pBI121 vector by XbaI and SacI. The constructed pHB-35S-AcPME2 and pBI121-35S-AcPME2 vectors were introduced into the agrobacterium EHA105.

For kiwifruit, the agrobacterium-mediated stable transformation of diploid “Hongyang” was performed according to the method described in the previous study (Wang et al. 2007). In short, fresh callus was soaked in EHA105 containing pHB-35S-AcPME2 for 15 min, then placed on selective media supplemented with 50 mg/L hygromycin to screen for resistant callus.

For tomato, micro-Tom were transformed by EHA105 containing pBI121-35S-AcPME2 according to the previous study (Li et al. 2016). The T-DNA insertion of transformed lines were verified by PCR using NPTII-specific primers and the expression of *AcPME2* were analyzed by RT-qPCR. The homozygous lines of T2 generation were used for phenotypic observation and stress tolerance evaluation.

For Arabidopsis, we did the stable transformation according to the floral dipping method (Clough and Bent 1998). The homozygous lines of T2 generation were used for further experiments.

### Ruthenium red staining

To detect the demethylesterified pectin, the untreated and treated roots were dyed with 0.01% (w/w) ruthenium red in a 50 mL EP tube for 5 min, and then washed with ddH_2_O (Wu et al. 2010).

### Immunostaining of pectin

The immunostaining was performed according to the method described in a previous study (Shi et al. 2021). The antibody LM19 was kindly provided by Dr. Chaowen Xiao in Sichuan University. The second antibody, Rabbit anti-Rat IgG (H&L), Fluorescein-5-isothiocyanate (FITC) conjugated, were purchased from Agrisera (#AS101523). FITC fluorescence was observed by using excitation 488 nm and emission collection 510 to 530 nm with a confocal microscope (Leica STELLARIS 5).

### Calcium ion fluorescence staining

The fluorescence probe (fluo-4/AM) were dissolved in the MS/2 liquid media to prepare the loading solution according to the previous research method (Qiu et al. 2020). For the untreated plants, roots were placed in the loading solution for 30 min in the dark. To stain the treated plants, roots were first immersed in the loading solution for 20 min and then placed in the loading solution containing 200 mm mannitol for 10 min. Then, the untreated and treated roots were washed with MS/2 liquid media quickly to remove the excess fluorescent dye and observed under confocal microscope (Qiu et al. 2020). Fluo-4/AM was observed by using excitation 488 nm and emission collection 510 to 520 nm with a confocal microscope (Leica STELLARIS 5).

### Plasmolysis of root hairs

To observe plasmolysis in Arabidopsis root hairs, Col-0 and the transgenic plants were treated in MS/2 liquid media containing 200 mm mannitol and the root hairs were continuously observed and photographed under microscope for 40 min. To observe plasmolysis in kiwifruit root hairs, the roots of “Hongyang” and transgenic plants were treated in MS/2 liquid media containing 300 mm mannitol.

### Transcriptome analysis

The 6-day-old seedlings of wild type Arabidopsis Col-0 and two transgenic lines (OE-7 and OE-17) were treated in MS/2 liquid media containing 200 mm mannitol for 15 and 25 min, respectively. Three biological replicates for each line under certain treatment were used for RNA sequencing. Total RNAs of 27 samples were extracted and sequenced by Shanghai Peisenol Biotech Co. (China). The differentially expressed genes (DEGs) were identified by using DESeq2 or edgeR software, with the parameters of absolute fold change ≥2 and false discovery rate <0.05.

### The reverse transcription-quantitative PCR (RT-qPCR)

The extraction of total RNAs and reverse transcription reaction were done as described previously (Li et al. 2016). qPCR was performed using the Step One Plus real-time PCR system. The relative expression level was calculated by the cycle threshold (Ct) 2^−ΔΔCt^ method. We used three biological replicates for all genotypes and treatments. Primers used here are listed in the Supplementary Table S3.

### The PME activity assay

The samples were homogenized in liquid nitrogen and dissolved in 50 mm sodium phosphate buffer (protease inhibitor cocktails, 500 mm NaCl, pH 7.5). The soluble protein fraction was extracted by centrifuging at 11,500g (20 min, 4℃). PME activity of three replicates was immediately measured after extraction, according to the previous description (Grsic-Rausch and Rausch 2004). The reaction was carried out in a reaction mixture (110 *μ*L), containing 10 *μ*L of protein extract (sample), 8 *μ*L of 0.5% (w/v) pectin (P9135 Sigma), 894 *μ*L NAD^+^ (4 mm), 0.35 U formaldehyde dehydrogenase (F1879 Sigma), 1 U alcohol oxidase (A2404 Sigma, pH 7.5), and 4 *μ*L of recombinant pectin esterase (P5400 Sigma) as positive control, or 10 *μ*L of phosphate buffer as negative control. PME activity was determined at 340 nm in a UV/Vis spectrophotometer for continuous recording. One PME unit was defined by 1 *μ*mol NADH formation/min.

### Accession numbers

Sequence data of *AcPME2* can be found in Kiwifruit Genome Database (http://kiwifruitgenome.org/) under accession numbers (Actinidia06151). The raw data of RNA sequencing was uploaded to the NCBI with the registration number PRJNA1087684.

## Supplementary Material

kiae475_Supplementary_Data

## Data Availability

The RNA sequencing data have been deposited at Sequence Read Archive database in NCBI and the accession numbers is PRJNA1087684. The data and materials included in the study are available from the corresponding authors upon request.

## Supplementary data

The following materials are available in the online version of this article.

**Supplementary Figure S1.** Protein sequence alignment of AcPME2 and AtPME34.

**Supplementary Figure S2.** A*c*PME2 overexpression increased the cell size.

**Supplementary Figure S3.** A*c*PME2 overexpression increased tomato fruit size.

**Supplementary Figure S4.** Osmotic stress enhanced pectin demethylesterification and calcium accumulation in roots of AcPME2-OE kiwifruit.

**Supplementary Figure S5.** Osmotic stress induced plasmolysis in kiwifruit.

**Supplementary Figure S6.** Exclusion of calcium delayed the stress-induced plasmolysis of root hairs.

**Supplementary Figure S7.** RNA-seq analysis of Col-0 and AcPME2-OE plants in response to osmotic stress

**Supplementary Table S1.** All the DEGs identified by RNA seq.

**Supplementary Table S2.** The stress-responsive genes affected by *AcPME2* overexpression.

**Supplementary Table S3.** Primers used in this study.

## Dive Curated Terms

The following phenotypic, genotypic, and functional terms are of significance to the work described in this paper:

RRTF1 Gramene: AT4G34410
RRTF1 Araport: AT4G34410

HSP70 Gramene: AT3G12580
HSP70 Araport: AT3G12580

THE1 Gramene: AT5G54380
THE1 Araport: AT5G54380

FER Gramene: AT3G51550
FER Araport: AT3G51550

UGE3 Gramene: AT1G63180
UGE3 Araport: AT1G63180

LOX4 Gramene: AT1G72520
LOX4 Araport: AT1G72520

DREB2A Gramene: AT5G05410
DREB2A Araport: AT5G05410

ruthenium red CHEBI: CHEBI:34956
