# Supplementary material for: Chromosome doubling increases PECTIN METHYLESTERASE 2 expression, biomass, and osmotic stress tolerance in kiwifruit
Source: Plant Physiol. 2024 Sep 9;196(4):2841–55. doi: 10.1093/plphys/kiae475 (PMC11637999; doi:10.1093/plphys/kiae475)
Supplement: kiae475_Supplementary_Data [file kiae475_supplementary_data.zip › Supplemental Figures.pdf]

```
AcPME2  1    ---------------------------------------------MGYGRLGKHEPEPSQPEPLTGQPDYNM-----SPP  30
AtPME34 1    MTRVPPIITTHSPYILHIQSFTHIYTTQSHILYNVNQKRTKPRNKMGYERLGPSGATGSVTTSTTAPILNQVSTSEQPE  80
                                                          TM

AcPME2  31   RTTKRTKLKLL---LFFSFAFILASAVSVSLLIKFRTTGSGESEAAIRARPTIAISRTCSRTRYPTLCVNSLLDFPGSHT  107
AtPME34 81   NNNRRSKKKLVVSSIVLAISLILAAAIFAGVRSRLKLN---QSVPGLARKPSQAISKACELTRFPELCVDSLMDFPGSLA  157

AcPME2  108  A-SESQLAHITMNVTLQRLGKALYFSSAISHFEMSSLVRSAYEDCLELLADSVSHLSRSLNSVGST----QDVLTWLSAA  182
AtPME34 158  ASSSKDLIHVTVNMTLHHFSHALYSSASLSFVDMPPRARSAYDSCVELLDDSVDALSRALSSVVSSSAKPQDVTTWLSAA  237
                                    PMEI (PRE-PRO region)
AcPME2  183  LTNQDTCTDGLEEVR-GPVRNVMEERLRDLGELVSNCLAIYAAASGGRNEDFSGVPIGNRRRMLAAGGSSGGVPRWLSRR  261
AtPME34 238  LTNHDTCTEGFDGVDDGGVKDHMTAALQNLSELVSNCLAIFSASHDG--DDFAGVPIQNRR-LLGVEEREEKFPRWMRPK  314

AcPME2  262  ERVLLDMPVAAIQADIVVSKDGNGSVKTIAEAIKKAPEYSTRRTIIYVKAGRYEEDNLKVGRKKTNLMFIGDGKGKTIIS  341
AtPME34 315  EREILEMPVSQIQADIIVSKDGNGTCKTISEAIKKAPQNSTRRIIIYVKAGRYEENNLKVGRKKINLMFVGDGKGKTVIS  394

AcPME2  342  GGKSIFDNMTTFHTASFAATGSGFIARDITFENWAGPSKHQAVALRVGADHAVVYRCNIIGYQDTLYVHSQRQFFRECDI  421
AtPME34 395  GGKSIFDNITTFHTASFAATGAGFIARDITFENWAGPAKHQAVALRIGADHAVIYRCNIIGYQDTLYVHSNRQFFRECDI  474
                                    PME (Mature region)
AcPME2  422  YGTVDFIFGNAAVVFQNCSIYARKGLPGQKNTVTAQNRKDPNQNTGISIHACRIMATSDLMPMKGNYSTFLGRPWKPYSR  501
AtPME34 475  YGTVDFIFGNAAVVLQNCSIYARKPMDFQKNTITAQNRKDPNQNTGISIHASRVLAASDLQATNGSTQTYLGRPWKLFSR  554

AcPME2  502  TVVMLSHIGDHVHPRGWLEWNTSNFALDTLYYGEYLNYGPGSALSQRVKWLGYRVINSTVEASQFTVAQFIFGSSWLPST  581
AtPME34 555  TVYMMSYIGGHVHTRGWLEWNTT-FALDTLYYGEYLNSGPGSGLGQRVSWPGYRVINSTAEANRFTVAEFIYGSSWLPST  633

AcPME2  582  GVAFLAGLSL   591
AtPME34 634  GVSFLAGLSI   643
```

Supplemental Figure 1. Protein sequence alignment of AcPME2 and AtPME34

The alignment of AcPME2 (Actinidia06151) and AtPME34 (AT3g49220) was performed by using online tool (https://blast.ncbi.nlm.nih.gov/Blast.cgi). The domains of transmembrane (TM) indicated with orange line, PMEI (PRE-PRO region) with green line, and PME (Mature region) with black line were labeled according to the previous study about PME34 (Huang et al., 2017). The red and blue letters indicate the identical and similar amino acids, respectively.

Supplemental Figure 2. ***AcPME2*** **overexpression increased the cell size.**

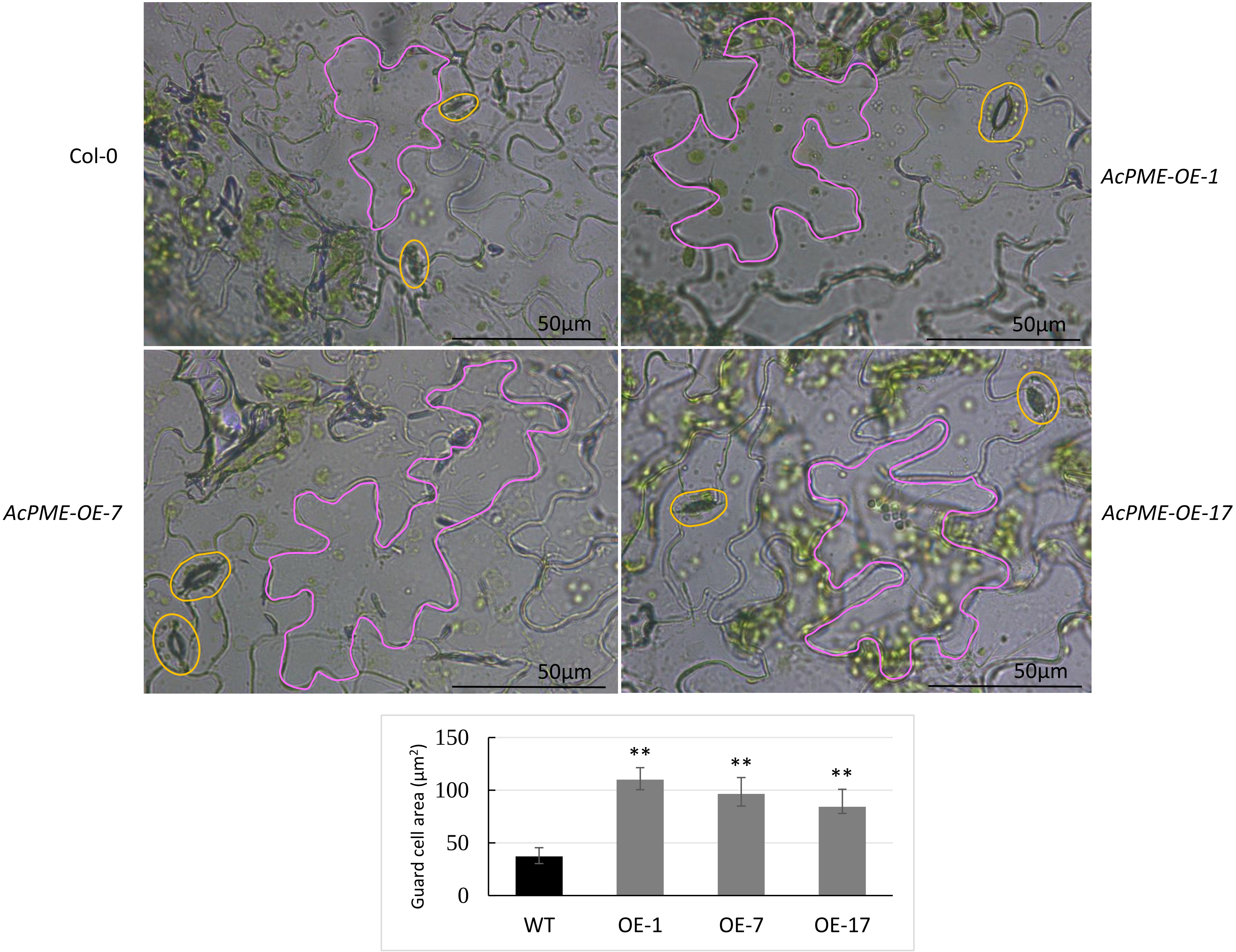

Supplemental Figure 2. ***AcPME2*** **overexpression increased the cell size.**

The microscope observation of epidermal cells of 3-week-old plant leaves of Col-0, AcPME2-OE-1, -7, and -17 grew in the soil. The single epidermal cell was highlighted by the magenta line. The guard cells were highlighted by yellow lines. The guard cell area were calculated by using ImageJ. Mean±SD were obtained from three independent experiments (n=12). “**” indicates the significant difference $p < 0.001$.

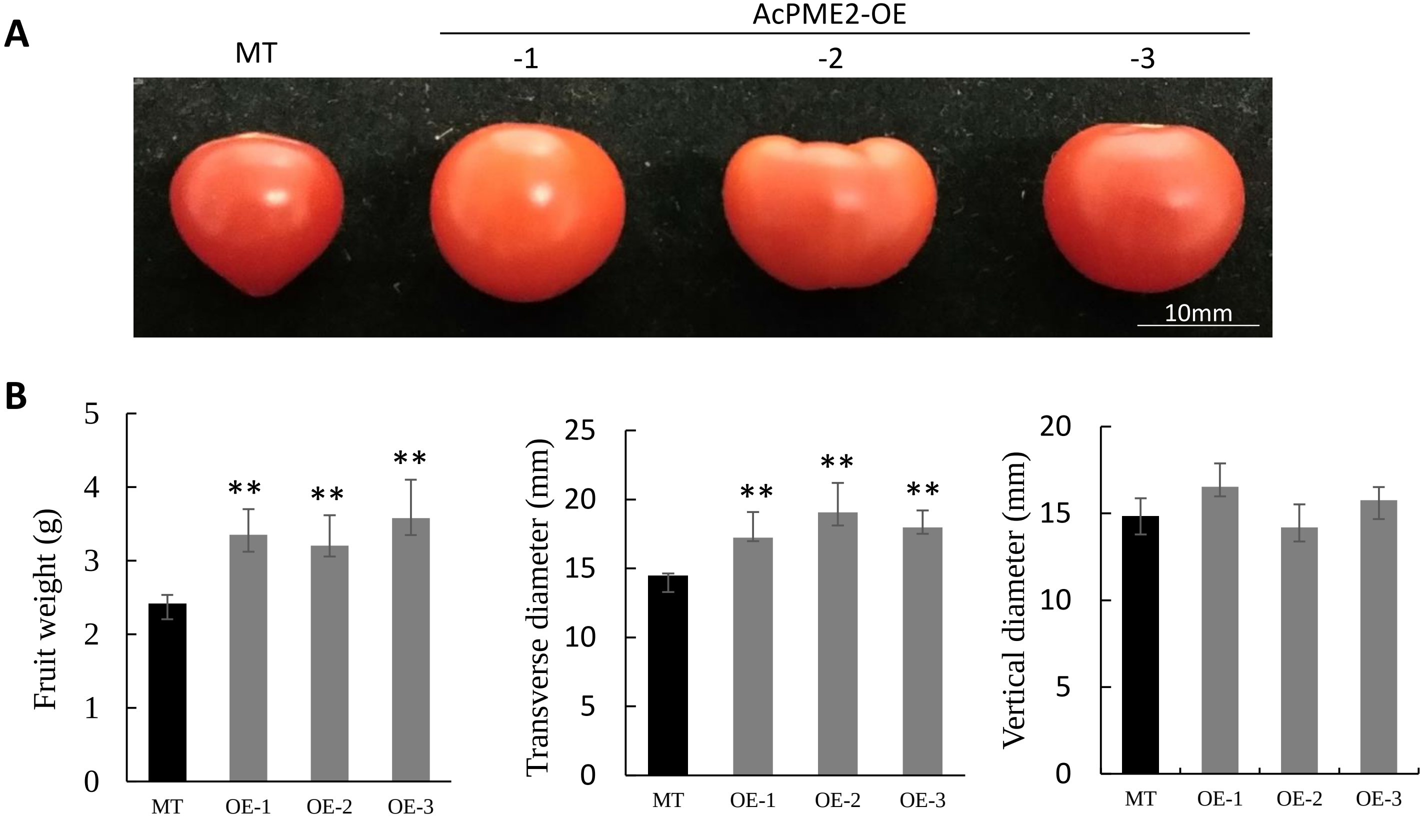

Supplemental Figure 3. ***AcPME2*** **overexpression increased the size of tomato fruit.**
(A) The ripe fruit of wild type tomato (micro-Tom, MT), AcPME2-overexpressing (-OE) lines. Bar=10mm. (B) The quantitative measurements of fruit weight, transverse and vertical diameter of above-mentioned lines. Mean$\pm$SD were obtained from three independent experiments ($n=10$). "**" indicates the significant difference $p < 0.001$.

Supplemental Figure 4. Osmotic stress enhanced pectin demethylesterification and calcium accumulation in roots of AcPME2-OE kiwifruit.

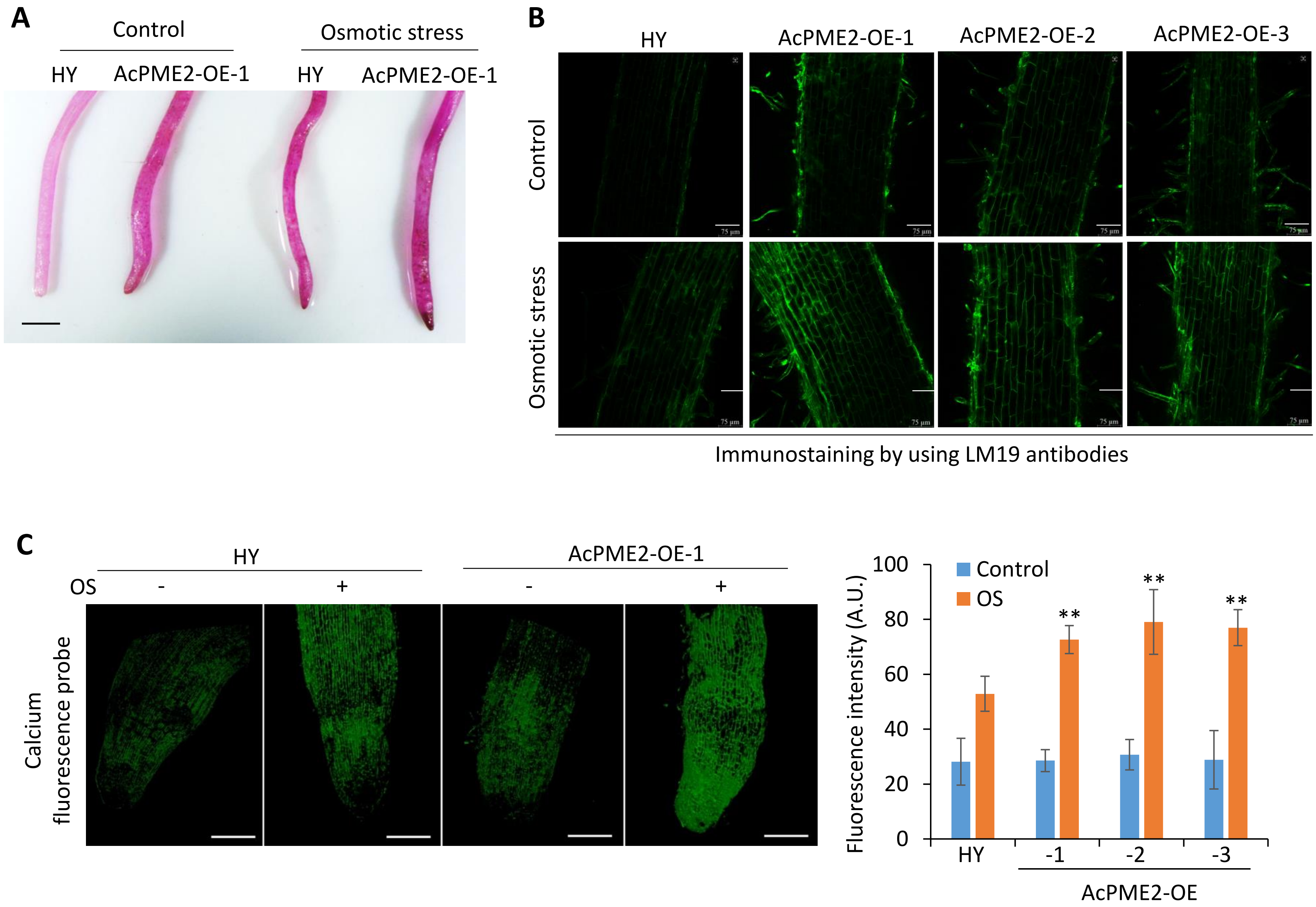

Supplemental Figure 4. Osmotic stress enhanced pectin demethylesterification and calcium accumulation in roots of AcPME2-OE kiwifruit.

(**A**) The ruthenium red staining of roots of diploidy ‘Hongyang’ (HY) and AcPME2-OE-1 treated in liquid MS/2 media without (control) or with 300mM mannitol for 10 min (osmotic stress, OS). Bar=1cm. (**B**) The immunostaining of roots of diploidy ‘Hongyang’ (HY) and AcPME2-OE plants treated without (control) and with 300mM mannitol for 10 min (osmotic stress) were performed using LM19 antibodies that recognize the demethylesterified pectin. Bar=75 μm. (**C**) The confocal microscope observation of calcium fluorescence probe (fluo-4/AM) in the roots of HY and AcPME2-OE lines (-1, -2, and -3) treated without (control, -) or with 200mM mannitol for 10 min (OS, +). The fluorescence intensity was quantitatively measured by using ImageJ. Mean±SD were obtained from three biological replicates (n=3). “**” indicate the significant difference at $p < 0.001$. Bar=150 μm.

Supplemental Figure 5. Osmotic stress induced plasmolysis in kiwifruit.

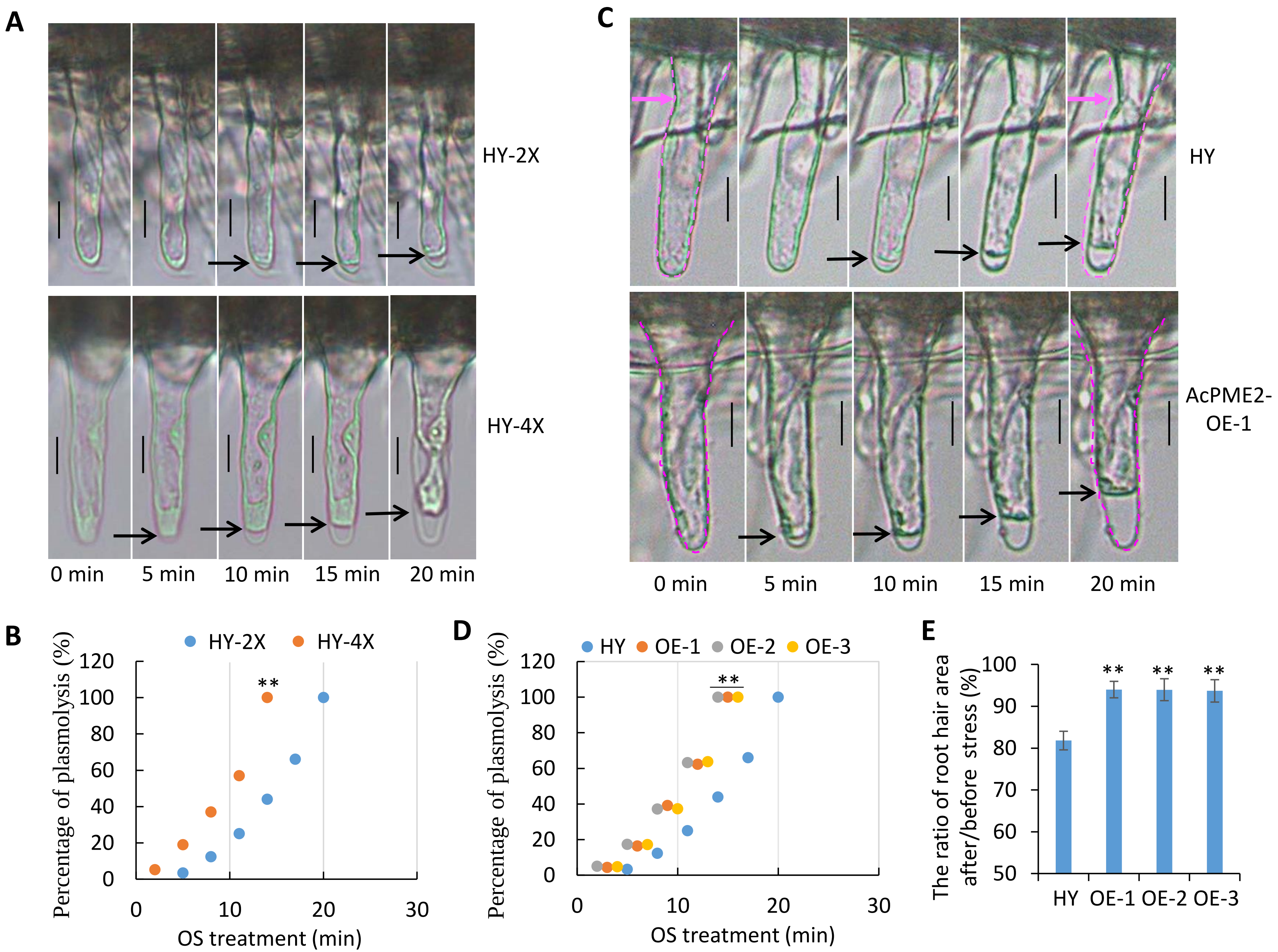

Supplemental Figure 5. Osmotic stress induced plasmolysis in kiwifruit.

Microscope observation of plasmolysis in the root hairs of diploid and tetraploid ‘Hongyang’ (HY-2X and HY-4X) (**A** and **B**), HY and AcPME2-OE lines (**C** and **D**) treated in the liquid MS/2 media containing 300 mM mannitol for 30 min. Bar=15μm. Black arrows indicated the plasmolysis. Magenta dashed lines in (**C**) showed the shapes of root hairs before osmotic stress and magenta arrows indicated the cell wall collapse. (**B** and **D**) Percentage of plasmolysis in the root hairs of above-mentioned lines with the same treatment. For each line, more than 20 root hairs from three independent experiments were observed and accounted for the percentage. “**” indicate the significant difference at $p < 0.001$. (**E**) The ratio (percentage) of root hair area after/before osmotic stress (300mM mannitol) treatment for 30 min. For each line, 15 root hairs from three independent experiments were observed and their areas were measured by ImageJ. The significant difference is calculated by comparing diploid HY with transgenic lines and indicated by “**” ($p < 0.001$).

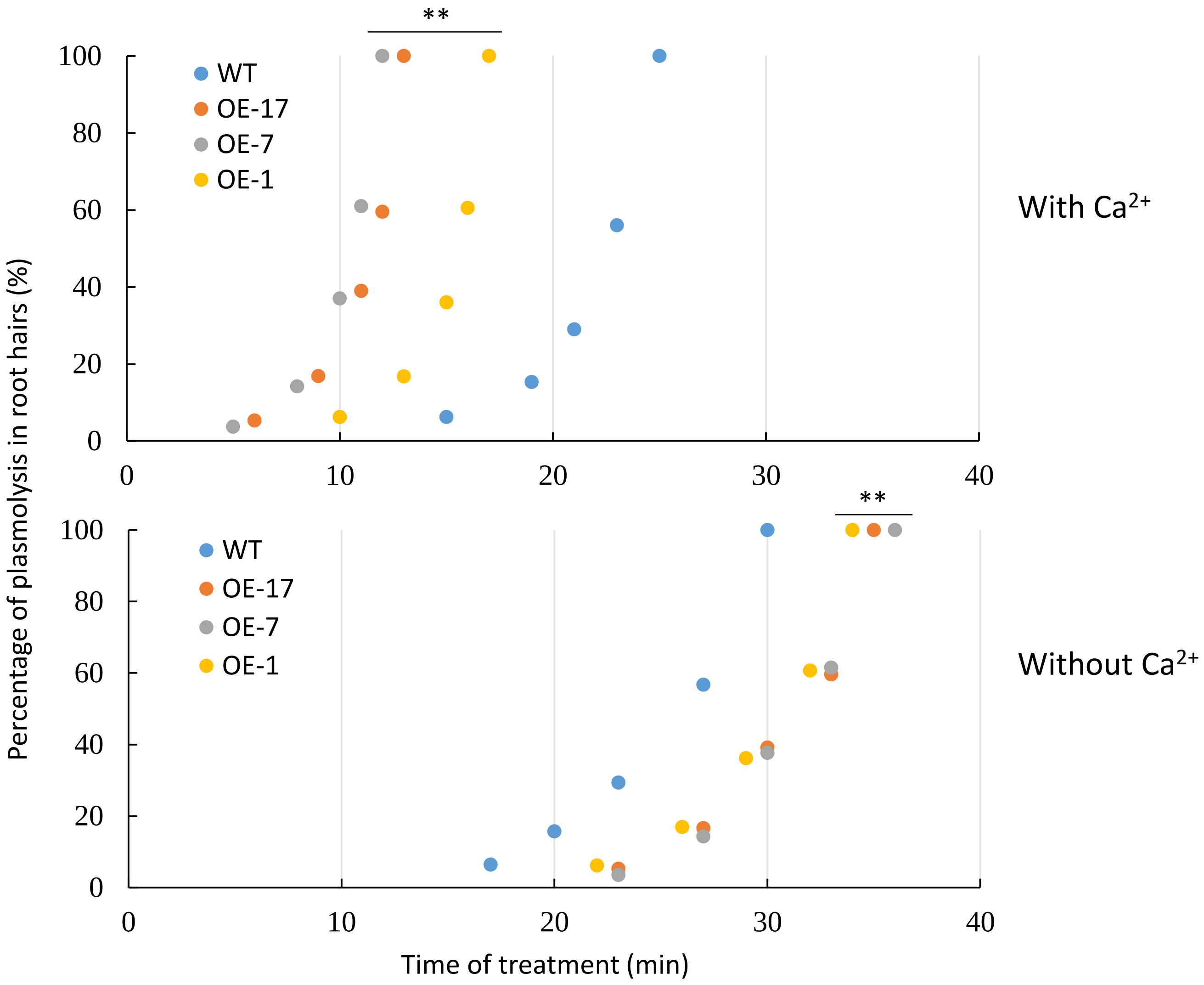

Supplemental Figure 6. Exclusion of calcium delayed the stress-induced plasmolysis of root hair. Percentage of plasmolysis in the root hairs of Col-0, AcPME2-OE-1, -7, and -17 treated in the liquid MS/2 media with or without Ca^{2+} containing 200 mM mannitol for 40 min. For each line, more than 20 root hairs from three independent experiments were observed and accounted for the percentage. "**" indicate the significant difference at $p < 0.001$.

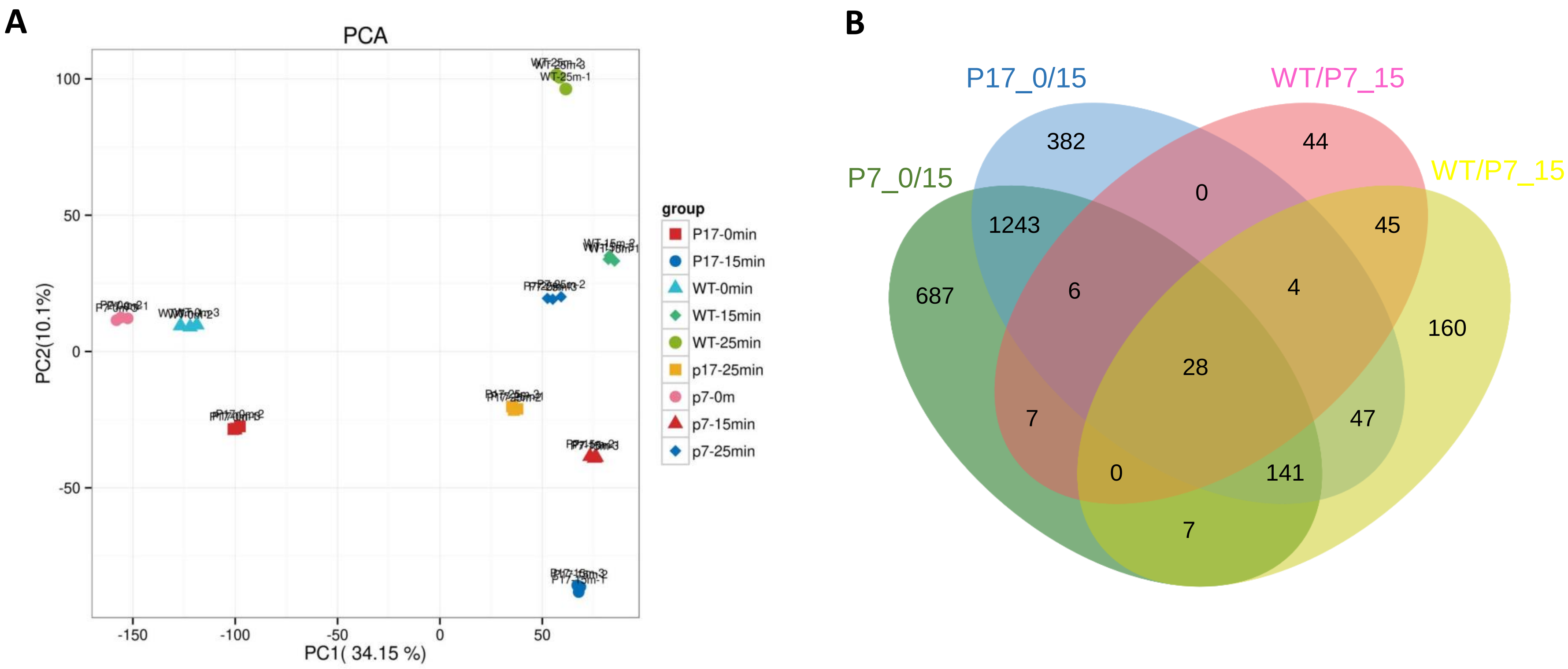

Supplemental Figure 7. RNA-seq analysis of Col-0 and AcPME2-OE plants in response to osmotic stress
(A) The principal component analysis (PCA) of three biological replicates of WT (Col-0), AcPME2-OE-7 (P7), and AcPME2-OE-17 (P17) roots treated in the liquid MS/2 containing 200mM mannitol for 0, 15, and 25 min, respectively. (B) The venn diagram of the differentially expressed genes (DEGs) between WT/P7 _15min, WT/P17_15, P7_0/15, and P17_0/15. The number of DEGs was showed.
