# Supplementary material for: Chromosome doubling increases PECTIN METHYLESTERASE 2 expression, biomass, and osmotic stress tolerance in kiwifruit
Source: Plant Physiol. 2024 Sep 9;196(4):2841–55. doi: 10.1093/plphys/kiae475 (PMC11637999; doi:10.1093/plphys/kiae475)
Supplement: kiae475_Supplementary_Data [file pp.00613.2024-s09.pdf › pp.00613.2024-s09.pdf]

Plant Physiology®

Chromosome doubling-induced high expression of AcPME2 leads to the increased biomass and stress tolerance in kiwifruit

Yanyan Zhu, Xinlei Wang, Yan He, Yajing Liu, Runze Wang, Yongsheng Liu, and Songhu Wang

Review Timeline:

Submission Date:	26-Apr-2024
Editorial Decision:	30-May-2024
Revision Received:	22-Jul-2024
Accepted:	09-Aug-2024

30-May-2024

Dr. Songhu Wang
Anhui Agricultural University
Hefei, Anhui
China

RE: Chromosome doubling-induced high expression of AcPME2 leads to the increased biomass and stress tolerance in kiwifruit

Dear Dr. Wang:

We have received reviews of your manuscript, which you may view at the end of this letter. On the basis of the advice received and my own evaluation, I believe that, pending revision, this article may ultimately be acceptable for publication in Plant Physiology.

In particular, please pay more attention to details.

When you are ready to submit your revision, click the below link and find the red arrow under your Author Tasks heading for this revision submission.

If you want to visit this version of the manuscript, you may click the link below and then click on the folder that reads "Post Decision Manuscripts".

Link Not Available

When uploading the revised version of this article, please be sure to include as complete an answer as possible to the referees' criticisms during the first round of reviews in the "response to reviewers" section.

Plant Physiology considers a revision for up to 60 days after a manuscript has been provisionally accepted; limited extension of this timeframe is at the discretion of the Monitoring Editor. A reminder notice will be sent to you 2 weeks before the deadline, at which time you may choose to contact the editorial office to request an extension; if so, please provide a justification for your request. After the expiration of this deadline, the article would need to be submitted as a resubmission and would be handled as such.

Thank you very much for giving us an opportunity to review this work. I look forward to receiving the next version.

Sincerely,

Editor
Plant Physiology

Want to add this revision deadline to your calendar? Click below!

-- Reviewer comments:
Reviewer #1 (Comments for the Author):

This manuscript focuses on functional characterization of gene AcPME2 from kiwifruit. Authors examined its role in increasing biomass and stress tolerance. Overexpression of AcPME2 in various species, including Arabidopsis, tomato, and kiwifruit, led to increased biomass and improved osmotic stress responses. The study explores how AcPME2 influences cell wall stiffness through its regulation of pectin demethylesterification and calcium accumulation, enhancing plant resilience under osmotic stress. This study highlights the potential of genetic manipulation in crop breeding for stress resilience and biomass enhancement.

The paper is well-written. The introduction is very informative and clear, with sufficient citation of latest research findings in this area. The method section is detailed. The results effectively demonstrate the role of AcPME2 in modulating plant responses to stress through cellular mechanisms, supported by both physiological and molecular data. The discussion appears to thoroughly address the implications of the findings, linking the gene AcPME2 to increased biomass and stress tolerance. Authors elucidate and discuss the role of AcPME2 in modifying cell wall stiffness through pectin demethylesterification and calcium accumulation.
Other Comments:
In the abstract, please indicate the source of AcPME2
Ln 27Upon short-term stress treatment" please indicate what stress here.
Ln 33 "under stress treatment" be specific with what stress.
Ln 46 underlying mechanisms, underlying the colchicine induced chromosome doubling? The cellular mechanism is relatively clear. It targets the organization of the microtubule system during cell division. By disrupting microtubule formation, colchicine prevents proper chromosome segregation, leading to polyploidization. Here should be molecular mechanisms.
Ln 101, please indicate what stress do you simulate with 400nM mannitol
Figure 1 B and C, please indicate what significance you measure, between treatment within 2x or 4X or between 2x and 4x within the same treatment.
Fig2 legend: MS/2 media should be 1/2 MS.
Fig.3 A, why is the AcPME2 in red? Fig.4A the bar is in blue. Please make sure the format to be consistent.
Ln 146, change HY plants to HY wild type plant
Ln 147 that of AcPME2-OE lines >>>> those of AcPME2-OE lines

Fig 5 and Fig.6 legend title should have what species has been used.

Fig.5B what difference are results in Fig.5B and Fig. 2B

Figure 5, what is the scale size in the image.
Ln202 than that of Col-0>>> than those of Col-0
In the discussion section, authors may elaborate more on why the faster plasmolysis caused by Ca+ accumulation in the AcPME2 overexpression line leads to stronger osmotic stress tolerance.

Reviewer #2 (Comments for the Author):

The work describes how the induction of high expression of AcPME2 by chromosome doubling leads to increased biomass and tolerance to osmotic stress in kiwifruit, which is innovative to some extent. The article generates a large amount of overexpression and mutant materials to verify the function of AcPME2, including Arabidopsis, tomato, and kiwifruit. However, the experiments related to resistance only cover osmotic stress, making the title's claim about resistance too general. The article also has some other minor issues. Modifications and improvements are suggested, with the specific issues as follows:
1. The significance analysis does not clearly indicate which is the control. It appears that comparisons are made with the same color blocks, which needs to be clarified.
2. Line 108-109, was it only AcPME2 expression that changes significantly and how was it screened?
3. In Fig. 2C, it is suggested to mark the tips of the root hairs for clearer observation.
4. In Fig. 5A, it is recommended to add other methods for detecting the degree of pectin demethylesterification, such as immunostaining.
5. In Fig. 5C, co-localization with cell wall probes is suggested to prove the presence of calcium ions in the cell wall.
6. Both Fig. 5A and Fig. 6 lack scales.
7. Regarding the increase in cell wall hardness, can direct experimental data be provided to support this?
8. The article mentions the impact of PME2 on biomass and stress tolerance, but the subsequent results only delve into stress tolerance. For biomass, only phenotypic observations are provided. Has transcriptome data been analyzed for biomass-related genes?

Reviewer #1 (Comments for the Author):

This manuscript focuses on functional characterization of gene AcPME2 from kiwifruit. Authors examined its role in increasing biomass and stress tolerance. Overexpression of AcPME2 in various species, including Arabidopsis, tomato, and kiwifruit, led to increased biomass and improved osmotic stress responses. The study explores how AcPME2 influences cell wall stiffness through its regulation of pectin demethylesterification and calcium accumulation, enhancing plant resilience under osmotic stress. This study highlights the potential of genetic manipulation in crop breeding for stress resilience and biomass enhancement.

The paper is well-written. The introduction is very informative and clear, with sufficient citation of latest research findings in this area. The method section is detailed. The results effectively demonstrate the role of AcPME2 in modulating plant responses to stress through cellular mechanisms, supported by both physiological and molecular data. The discussion appears to thoroughly address the implications of the findings, linking the gene AcPME2 to increased biomass and stress tolerance. Authors elucidate and discuss the role of AcPME2 in modifying cell wall stiffness through pectin demethylesterification and calcium accumulation.

Other Comments:

In the abstract, please indicate the source of AcPME2

Respond: Thank you for your suggestion. We added the relative information in the Abstract.

Ln 27Upon short-term stress treatment" please indicate what stress here.

Respond: Thank you for your suggestion. We revised the manuscript accordingly.

Ln 33 "under stress treatment" be specific with what stress.

Respond: Thank you for your suggestion. We revised the manuscript accordingly.

Ln 46 underlying mechanisms, underlying the colchicine induced chromosome doubling? The cellular mechanism is relatively clear. It targets the organization of the microtubule system during cell division. By disrupting microtubule formation, colchicine prevents proper chromosome segregation, leading to polyploidization. Here should be molecular mechanisms.

Respond: Thank you for the comment. Actually, “the underlying mechanisms” here was used to indicate why the chromosome doubling can lead to the increased cell size and yield. To avoid the confusion, we deleted the sentence from the revised manuscript.

Ln 101, please indicate what stress do you simulate with 400nM

mannitol

Figure 1 B and C, please indicate what significance you measure, between treatment within 2x or 4X or between 2x and 4x within the same treatment.

Respond: Thank you for your suggestion. We revised the manuscript accordingly. The significance is calculated by comparing the columns with the same colors between 2x and 4x plants.

Fig2 legend: MS/2 media should be 1/2 MS.

Respond: We revised as your suggestion.

Fig.3 A, why is the AcPME2 in red? Fig.4A the bar is in blue. Please make sure the format to be consistent.

Respond: We revised as your suggestion.

Ln 146, change HY plants to HY wild type plant

Respond: We revised as your suggestion.

Ln 147 that of AcPME2-OE lines >>>> those of AcPME2-OE lines

Respond: We revised as your suggestion.

Fig 5 and Fig.6 legend title should have what species has been used.

Respond: We revised as your suggestion.

Fig.5B what difference are results in Fig.5B and Fig. 2B

Respond: Fig. 2B showed the PME activities of whole plants, while Fig. 5B showed those of roots. We emphasized the difference in the figure legends.

Figure 5, what is the scale size in the image.

Respond: We added the scale in the pictures of Figure 5 and 6.

Ln202 than that of Col-0>>> than those of Col-0

Respond: We revised as your suggestion.

In the discussion section, authors may elaborate more on why the faster plasmolysis caused by Ca+ accumulation in the AcPME2 overexpression line leads to stronger osmotic stress tolerance.

Respond: Thank you for the comment. We added relative information in the Discussion, which is highlighted in the red.

Reviewer #2 (Comments for the Author):

The work describes how the induction of high expression of AcPME2 by chromosome doubling leads to increased biomass and tolerance to osmotic stress in kiwifruit, which is innovative to some extent. The article generates a large amount of overexpression and mutant materials to verify the function of AcPME2, including Arabidopsis, tomato, and kiwifruit. However, the experiments related to resistance only cover osmotic stress, making the title's claim about resistance too general.

Respond: Thank you for the comments. We changed the title of our manuscript into "Chromosome doubling-induced high expression of AcPME2 leads to the increased biomass and osmotic stress tolerance in

kiwifruit". Also, we specified the osmotic stress in the Abstract.

The article also has some other minor issues. Modifications and improvements are suggested, with the specific issues as follows:

1. The significance analysis does not clearly indicate which is the control. It appears that comparisons are made with the same color blocks, which needs to be clarified.

Respond: Thank you for the comment. We revised all the figure legends accordingly.

2. Line 108-109, was it only AcPME2 expression that changes significantly and how was it screened?

Respond: In our previous study (Zhu et al., 2021), the transcriptomic analysis revealed that more than ten *PME* genes were up-regulated in the tetraploid HY plants. Among those genes, *AcPME2* (Actinidia06151) showed the highest expression and most significant difference between diploid and tetraploid plants. Therefore, we chose the gene for the further functional characterization in this study.

3. In Fig. 2C, it is suggested to mark the tips of the root hairs for clearer observation.

Respond: Thank you for the suggestion. We marked the root tips with red or yellow lines in the revised Fig. 2C.

4. In Fig. 5A, it is recommended to add other methods for detecting the degree of pectin demethylesterification, such as immunostaining.

Respond: Thank you for the suggestion. We used the antibody LM19, which recognizes the demethylesterified pectin, to perform immunostaining. The results were showed in the Fig. 5C and Supplemental Fig. 4B of the revised manuscript and were consistent with that of ruthenium red staining.

5. In Fig. 5C, co-localization with cell wall probes is suggested to prove the presence of calcium ions in the cell wall.

Respond: Thank you for the suggestion. We used the plasmolysis to confirm calcium is co-localized with cell wall. We extended the treatment of osmotic treatment (from 10 min to 25 min) to cause the plasmolysis in the root hairs. The fluorescence signals were still associated with cell walls when plasmolysis occurred, as indicated by the red arrows in the Fig. 6C of the revised manuscript. The shrinking plasma membrane also displayed very weak fluorescence signals (Fig. 6C). These results indicated that cell walls contain much more Ca^{2+} than plasma membrane under osmotic stress.

6. Both Fig. 5A and Fig. 6 lack scales.

Respond: We added the scale in the pictures of Figure 5 and 6.

7. Regarding the increase in cell wall hardness, can direct experimental data be provided to support this?

Respond: To directly visualize the increased cell wall stiffness in the AcPME2-OE plants, we used the higher concentrations of mannitol

(250mM) to perform the stress treatment. The cell wall collapse and deformation were clearly observed in the root hairs of Col-0 and *pme34* mutant, but not in those of AcPME2-OE plants (Fig. 8). These results indicated that AcPME2 overexpression significantly increased the cell wall stiffness under osmotic stress, which provided mechanical support for plant survival from the stress.

8. The article mentions the impact of PME2 on biomass and stress tolerance, but the subsequent results only delve into stress tolerance. For biomass, only phenotypic observations are provided. Has transcriptome data been analyzed for biomass-related genes?

Respond: Thank you for the comment. For the impact of PME on biomass, many previous studies have clearly demonstrated that PME-mediated demethylesterification contributes to cell wall loosening and cell extension by making homogalacturonans (HGs) available for HG-modifying enzymes, such as pectate lyases (Colin et al., 2023), or providing the platforms that anchor peroxidases to remodel plant cell wall (Moustacas et al., 1991; Francoz et al., 2019;). The cell wall loosening led to the increased cell size, which was also observed in the AcPME2-OE plants in this study. We believe that *AcPME2* uses the similar way as other *PME* genes to increase the cell size and thereby to increase biomass under control condition. As for the DEGs related to the biomass in our transcriptomic data, we speculate there might be some DEGs

related to biomass in our data, but they aren’t directly regulated by *AcPME2*. Therefore, these genes are not our concern.

Colin L, Ruhnow F, Zhu JK, Zhao CZ, Zhao Y, Persson S (2023) The cell biology of primary cell walls during salt stress. Plant Cell **35:** 201-217

Francoz E, Ranocha P, Le Ru A, Martinez Y, Fourquaux I, Jauneau A, Dunand C, Burlat V (2019) Pectin Demethylesterification Generates Platforms that Anchor Peroxidases to Remodel Plant Cell Wall Domains. Dev Cell **48:** 261-276 e268

Moustacas AM, Nari J, Borel M, Noat G, Ricard J (1991) Pectin methylesterase, metal ions and plant cell-wall extension. The role of metal ions in plant cell-wall extension. Biochem J **279 (Pt 2):** 351-354

09-Aug-2024
Dr. Songhu Wang
Anhui Agricultural University
Hefei, Anhui
China

MSID: PP2024-RA-00613R1
MS TITLE: Chromosome doubling-induced high expression of AcPME2 leads to the increased biomass and stress tolerance in kiwifruit

Dear Dr. Songhu Wang:

I am pleased to inform you that your revised manuscript "Chromosome doubling-induced high expression of AcPME2 leads to the increased biomass and stress tolerance in kiwifruit" has been accepted for publication in Plant Physiology. Your manuscript will be checked for consistency with journal formatting and image standards by one of our Science Editors, who will contact you shortly. They may have a number of queries and suggestions for you. Please keep in mind that they work to increase the online visibility of your article and its attraction for readers from the widest of backgrounds. You will have the opportunity to review any revisions, and I hope you will work constructively with them to get the most out of your research. You will also have the opportunity to review all changes when you receive the author proofs for your article.

Once validated for publication, your article will be sent to a Science Editor for a final review of your manuscript and source files. After your final files are uploaded, your article will appear in the next available issue and the online Preview version of your article will be posted after your signed Copyright and Checklist forms and final source files are received.

Please be sure to include the following in your manuscript file:
Harvard style citations
List of all author contributions and funding information at the beginning of manuscript after the titles and author list.
List all supplemental materials associated with the article, with titles, at the end of the ms, before the references and acknowledgments section.
Complete Figure Legends for all figures appearing in the article listed before the Literature Cited.

Thank you and congratulations on your Plant Physiology paper!

Sincerely,
Editor
Plant Physiology

=====
FIRST AUTHOR PROFILES
=====

We recognize the hard work that goes into being First Author of a Plant Physiology paper by publishing a "First Author Feature" for each First Author alongside your paper.

If the first author(s) would like to submit information for a feature on Facebook and Twitter, fill out the form by clicking on the link below:

Plant Physiology First Author Feature Form: https://forms.office.com/r/ZBCGZfhm8F
If you cannot access the form through the link above, please contact Rachel at rbelsky@aspb.org.

To upload an image of yourself to accompany your profile, please put your photo in the correct journal folder at the link below. You must label your image: Author First Name Last Name Article Title.

First Author Profile Image Dropbox: https://www.dropbox.com/scl/fo/xz029p7qjr2jml2rp2kmq/h?dl=0&rlkey=6fkxcaqmxx35ap152b2osy1fz

=====
IMPORTANT REMINDER: PEER REVIEW REPORTS
=====

If you opted to publish a peer review report along with your article during the original submission process, it will be prepared by the editorial staff and publicly posted with your manuscript, inside the zip file that contains any other supplemental material. As a reminder, the peer review report is a public record of all comments from editors and reviewers, as well as your prior responses, as you received them in the decision letters for each draft of your manuscript. If you agreed to publish this report and have changed your mind, or are not sure if you selected this option, please contact the editorial office as soon as possible before signing the license agreement from our publisher.

-- Reviewer comments:
Reviewer #1 (Comments for the Author):

Authors of this manuscript have addressed all of my concerns. The revised manuscript is greatly improved.

Reviewer #2 (Comments for the Author):

Thank you for your reply. While your answers addressed my questions thoroughly, I have a few suggestions regarding writing conventions and formatting. For instance, "400mM" should be corrected to "400 mM" in line 103, and this correction should be applied consistently throughout the document. Please thoroughly check and revise the entire manuscript accordingly.
